# Characterization of the foreign body response of titanium implants modified with polyphenolic coatings

**DOI:** 10.1002/jbm.a.37377

**Published:** 2022-02-26

**Authors:** Florian Weber, Huy Quang Quach, Mathias Reiersen, Sadaf Yosef Sarraj, Dyala Nidal Bakir, Victor Aleksander Jankowski, Per H. Nilsson, Hanna Tiainen

**Affiliations:** ^1^ Department of Biomaterials Institute of Clinical Dentistry, University of Oslo Oslo Norway; ^2^ Department of Immunology Institute of Clinical Medicine, University of Oslo Oslo Norway; ^3^ Department of Chemistry and Biomedical Sciences Linnaeus University Kalmar Sweden

**Keywords:** antioxidant, blood, fibroblasts, pyrogallol, surface modification, tannic acid, wound healing

## Abstract

The foreign body response is dictating the outcome of wound healing around any implanted materials. Patients who suffer from chronic inflammatory diseases and impaired wound healing often face a higher risk for implant failure. Therefore, functional surfaces need to be developed to improve tissue integration. For this purpose, we evaluated the impact of surface coatings made of antioxidant polyphenolic molecules tannic acid (TA) and pyrogallol (PG) on the host response in human blood. Our results showed that although the polyphenolic surface modifications impact the initial blood protein adsorption compared to Ti, the complement and coagulation systems are triggered. Despite complement activation, monocytes and granulocytes remained inactivated, which was manifested in a low pro‐inflammatory cytokine expression. Under oxidative stress, both coatings were able to reduce intracellular reactive oxygen species in human gingival fibroblasts (hGFs). However, no anti‐inflammatory effects of polyphenolic coatings could be verified in hGFs stimulated with lipopolysaccharide and IL‐1β. Although polyphenols reportedly inhibit the NF‐κB signaling pathway, phosphorylation of NF‐κB p65 was observed. In conclusion, our results indicated that TA and PG coatings improved the hemocompatibility of titanium surfaces and have the potential to reduce oxidative stress during wound healing.

**Abbreviations**
ABTS: 2,2′‐azino‐bis(3‐ethylbenzothiazoline‐6‐sulfonic acid
DMEM: Dulbecco's modified Eagles medium
F1 + 2: prothrombin fragment 1 + 2
GA: gallic acid
hGF: human gingival fibroblast
IgG: immunoglobulin G
IL: interleukin
LDH: lactate dehydrogenase
LPS: (bacteria derived) lipopolysaccharide
MCP‐1: monocyte chemoattractant protein 1
NF‐κB: nuclear factor kappa B
PBS: phosphate buffered saline
PG: pyrogallol
PG 70: PG coating obtained at pH = 7.0
PGG: penta‐galloyl glucose
ROS: reactive oxygen species
QCM‐D: quartz crystal microbalance with dissipation monitoring
TA: tannic acid
TA 68: TA coating obtained at pH = 6.8
TA 78: TA coating obtained at pH = 7.8
TAT: thrombin‐antithrombin complex
TBHP: tert‐butyl hydroperoxide
TCC: terminal complement complex
TCP: tissue culture plastic
TEAC: trolox equivalent activity concentration

## 1 INTRODUCTION

Titanium (Ti) is a widespread material in medical applications due to its mechanical properties and corrosion resistance.^1^ Further, a firm tissue integration and low foreign body response has made titanium and its alloys a popular choice as material for bone anchored implants and cardiovascular applications.^2^ Particularly for transcutaneous and transmucosal implants, such as auricular or dental implants, their clinical success strongly relies on their integration in the surrounding hard and soft tissues.^3^ With the natural skin barrier impaired, infections caused by bacteria often lead to failure of the implant.^4^, ^5^ Commonly, challenges in tissue integration and wound healing processes have addressed the hard tissue, but efforts need to be made to also improve the soft tissue integration.^6^, ^7^

Wound healing around implants starts with the direct contact with blood. The immediate adsorption of plasma proteins onto the implant surface dictates the subsequent activation of immune cells, the complement system, and the coagulation cascade.^8^ Thus, control of the foreign body reaction towards implants results in faster wound healing and close contact between the peri‐implant tissue and the implant surface can therefore be established during the subsequent resolution of the inflammatory phase. Thereby, the entry of potential pathogenic microbes to the wound site and colonization of the implant surface can be prevented.^9^

A variety of different surface modification strategies have emerged to improve the tissue integration of titanium implants. With respect to dental implants, approaches to change the surface topography and its physical, chemical, and biological properties have been studied.^7^ The underlying aim is to modulate protein adsorption, cell responses, and the microbial colonization of the implant surface. Among the chemical and biological surface modifications, changes of surface charge and hydrophilic properties, or functionalization with different bioactive molecules have been explored.^10^ Recently, polyphenolic molecules have been suggested as potential candidates to tackle inflammation and reduce microbial colonization.^11^ Their anti‐inflammatory properties are attributed to their capability to reduce oxidative stress,^12^ which is based on catechol and gallol units. These structures are able to scavenge radicals and redox active ions.^13^ In addition, polyphenols can reduce inflammation via modulating cellular signaling pathways.^14^ In particular, inhibition of IκB kinase on the nuclear factor κB (NF‐κB) signaling pathway in macrophages has been reported for several polyphenolic molecules.^15^, ^16^, ^17^ Similarly, inhibition of pro‐inflammatory cytokine expression by polyphenols has been shown in gingival fibroblasts exposed to bacterial lipopolysaccharide (LPS).^18^ Therefore, a variety of polyphenolic molecules have been suggested as treatment options for patients with inflammatory periodontal diseases.^19^, ^20^

Since certain polyphenols are able to form surface coatings,^21^ this property may be harnessed for local delivery of active molecules in the peri‐implant environment. For example, Ti surfaces modified with dopamine, quercetin, curcumin, and tannic acid (TA) have shown osteopromotive and anti‐inflammatory effects on various cell types in vitro.^22^, ^23^, ^24^, ^25^, ^26^

Despite their potential to modulate the foreign body response, polyphenolic surface modifications have so far not been systematically investigated for early wound healing processes in soft tissues. Therefore, we evaluated the effect of TA and PG coatings on protein adsorption, blood coagulation and complement activation. Further, we investigated the anti‐inflammatory capacity and the cytocompatibility of polyphenolic coatings in both human gingival fibroblasts (hGFs) and human whole blood to establish their potential for an improved wound healing around titanium implants.

## 2 EXPERIMENTAL

### 2.1 Materials

Details for the used materials and assays described in the experimental setups listed below are given in the ESI.

### 2.2 Coating formation

Tannic acid and PG coating solutions were prepared at a concentration of 1 mg/ml. TA solutions contained 100 mM HEPES, 600 mM NaCl, 100 μM Si_aq_ at either pH = 6.8 or pH = 7.8, to investigate the effect of a different coating thickness and chemistry.^27^ PG solutions contained 100 mM HEPES, 100 mM MgCl_2_ at pH = 7.0.^28^ Grade IV titanium coins were aseptically coated in vials containing 10 ml of coating solution on a rocking platform at 30 rpm for 24 h (Figure S1). After the coating formation, the coins were washed and sonicated in sterile water to remove unbound molecules and precipitated particles. The coated surfaces are referred to as TA 68, TA 78, and PG 70 depending on the coating solution while the uncoated control surfaces are called Ti.

### 2.3 Plasma and protein adsorption

Lyophilized citrated human plasma was reconstituted with water and diluted to 25% (v/v) in 10 mM phosphate buffer supplemented with 137 mM NaCl (PBS) at pH = 7.0. Fibrinogen, bovine serum albumin (BSA), and IgG were diluted in PBS at 200 μg/ml. Adsorption was quantified using a quartz crystal microbalance with dissipation monitoring (QCM‐D, QSense E4, BiolinScientific) under initial flow of 100 μl/min for 5 min at 21°C, followed by monitoring of the adsorption at a flowrate of 10 μl/min. Polyphenolic coatings were performed for 2 h and rinsed with PBS for 15 min before plasma and protein adsorption. The viscoelastic properties of the protein layer were modeled as described in the supporting information.

### 2.4 Blood sampling and blood experiment design

Experiments with human blood were performed according to the ethical guidelines from the declaration of Helsinki with the approval of local ethical committee. Informed written consent was obtained from all blood donors. Blood from healthy human donors was obtained by forearm venipuncture using a 21‐gauge needle. The whole blood (4.5 ml) was collected in polypropylene tubes containing 0.5 ml of 500 μg/ml of the thrombin inhibitor lepirudin (Refludan®, Celgene, Uxbridge, UK) as anticoagulant. Immediately after collection, an aliquot of 400 μl of blood was taken and 64 μl of a stop solution (CTAD solution [0.08 M trisodium citrate, 11 M theophylline, 2.6 M adenosine, 0.14 M dipyridamole] and 0.14 M EDTA) was added to this aliquot. This blood sample was centrifuged at 3000 *g* for 30 min at 4°C. After centrifugation, plasma in the supernatant was collected and referred to as *T*_0_ sample. Meanwhile, 400 μl of blood was incubated with one coin each of the different surfaces in a rolling incubator at 37°C. Depending on detection markers, as described below, an aliquot of incubated blood was taken at different time points. Blood was collected from three donors and experiments with blood from each donor was performed in triplicates (*n*_e_ = 9).

#### 2.4.1 Complement activation

After 30 min incubation, aliquots of 80 μl of incubated blood as described earlier were transferred into polypropylene vials containing 12.8 μl of the stop solution, followed by centrifugation at 3000 *g* for 30 min at 4°C. After centrifugation, plasma was collected and stored at −80°C for further analysis. Activation of the complement system was quantified by the level of its activation products C4d, and terminal C5b‐9 complement complex or (TCC), using ELISA kits following the instruction provided by the manufacturer (see the ESI). The alternative pathway convertase, C3bBbP, was quantified using an in‐house ELISA assay.^29^ PBS and *Escherichia coli* (10^7^/ml) were used as negative and positive controls, respectively.

#### 2.4.2 Coagulation activation

Activation of the coagulation system was quantified by the level of the thrombin activation markers thrombin‐antithrombin complex (TAT) and prothrombin fragments F1 + 2 using ELISA kits following the instruction provided. Sample aliquots were taken 30 min after start of incubation, as described for the complement activation. PBS and *E. coli* (10^8^/ml) were used as negative and positive controls, respectively.

#### 2.4.3 Platelet activation

After 30 min of incubation, 40 μl blood was taken to detect platelet activation. After adding 6.4 μl of the stop solution, the platelets were stained for 30 min in the dark at 4°C with CD42a‐FITC, CD63‐PE‐Cy7, and CD62P‐PE. After lysing red blood cell with a fixative‐free lysis buffer (Introvigen™, Cat# HYL250), the platelets were resuspended and fixed in PBS containing 0.1% paraformaldehyde (PFA) and 0.1% bovine serum albumin (PBSA). Platelet activation was measured using an Attune NxT Acoustic Focusing Cytometer (Thermo Fisher Scientific). Platelets were gated as CD42a positive (CD42a+) population while their activation was measured by the expression of CD62P and CD63 markers on their surfaces. Data was analyzed with Flowjo software version 10.

#### 2.4.4 Granulocyte and monocyte activation

A 40 μl aliquot of incubated blood was taken after 30 min to quantify the activation of granulocytes and monocytes. After adding 6.4 μl of the stop solution, the cells were stained for 30 min in the dark at 4°C with CD45‐Pacific Orange, CD14‐PerCP, CD11b‐APC/Fire 750, and CD35‐Alexa Fluor 647. After lysing red blood cell with the fixative‐free lysis buffer, the cells were resuspended and fixed in PBSA. The expression of abovementioned markers was analyzed with Attune NxT Acoustic Focusing Cytometer. Granulocytes were gated as the population positive for both CD45 and CD15 (CD45+/CD15+), while monocytes were gated as the population positive for both CD45 and CD14, but negative for CD15 (CD45+/CD14+/CD15−). The activation of granulocytes and monocytes was evaluated by the expression of CD11b and CD35.

#### 2.4.5 Cytokine profile

After 4 h incubation, 25.6 μl of the stop solution was added to remaining 160 μl incubated blood. Plasma collected after centrifugation was analyzed for the levels of a panel of 27 cytokines using a MAGPIX multiplex system (Bio‐Rad Laboratories). The data were assessed by software optimized standard curves as generated using Human Standard I (Bio‐Rad) supplied with the multiplex kit.

### 2.5 Antioxidant capacity

#### 2.5.1 Dissolved polyphenolic molecules

The antioxidant capacity of polyphenolic molecules was quantified using the 2,2′‐azino‐bis(3‐ethylbenzothiazoline‐6‐sulfonic acid (ABTS) assay.^30^ 7 mM ABTS was mixed 1:1 with 2.45 mM K_2_(SO_4_)_2_ and left to react overnight to create the ABTS^+•^ radical. The solution was diluted with MilliQ water to an adsorption value of 0.7 at *λ* = 734 nm (Lambda25, PerkinElmer, using a quartz cuvette with 10 mm path‐length). Then 10 μl/ml of polyphenol solution was added to the ABTS solution, and the mixture was vortexed for 1 min. The absorbance was then measured after 5 min. The trolox equivalent antioxidant capacity (TEAC) was calculated from a calibration curve.

#### 2.5.2 Antioxidant capacity of coatings

The antioxidant capacity of polyphenolic coatings was quantified using the ABTS assay adapted for a microplate reader. Briefly, ABTS solution was prepared as described above and the absorption was adjusted to 1.0 for 200 μl in a 96‐well. Six coated Ti coins were incubated in ABTS solution at 1 ml/coin in the dark at room temperature (RT) with gentle shaking at 50 rpm. Then, 200 μl aliquots were taken at different time‐points and the absorbance at *λ* = 690 nm was measured.

### 2.6 Quantification of released polyphenols

The total dissolved polyphenol content was quantified using the Prussian Blue assay.^31^ In brief, MilliQ water was filtered thrice through activated charcoal to remove any iron contaminants. Six coated coins were incubated in filtered MilliQ water at 1 ml/coin at RT under gentle shaking at 50 rpm. Aliquots of 150 μl were then transferred to a 96‐well plate. To each well of 96‐well plate, 25 μl of 20 mM FeCl_3_ dissolved in 0.1 M HCl was added, followed by 25 μl of 16 mM K_4_Fe(CN)_6_. The color was left to develop for 5 min at RT under shaking before the absorbance at *λ* = 690 nm was measured. The concentration was calculated from a standard curve.

The release kinetics was quantified using a quartz crystal microbalance (QSense E4, BiolinScientific) in situ under constant flow of 100 μl/min. After equilibration of Ti sensors (QSX310) in coating buffer, rinsing buffer (10 mM citrate/phosphate buffer, 137 mM NaCl) was flown to obtain the buffer‐based change in frequency (Δ*F*) and dissipation (Δ*D*). TA and PG coatings were then deposited for 2 and 8 h, respectively. Before changing to rinsing buffer, the chamber was flushed with coating buffer to remove residual TA and PG molecules.

### 2.7 hGF cell culture

Human gingival fibroblasts (hGFs; Provitro) were routinely cultured at 37°C/5% CO_2_ in Dulbecco's modified Eagle's medium (DMEM), containing 4500 mg/ml glucose, 10% fetal bovine serum (FBS), 50 U/ml penicillin, 50 mg/ml streptomycin, and 2 mM GlutaMAX. Cells were subcultured before reaching confluence using trypsin/EDTA. Trypan blue stain was used to determine total and viable cell number. Cells were seeded at a density of 7 × 10^3^ cells/well in 96‐well plates, except for the comet assay (3.5 × 10^3^ cells/well) and intracellular reactive oxygen quantification (25 × 10^3^ cells/well in 48‐well plate). Experiments were performed with cells at passages ≤8 after isolation.

#### 2.7.1 Induction of inflammation in hGFs

Following 2 h incubation at 37°C/5% CO_2_ to allow cell adhesion, cells were washed with PBS and inflammation was induced by incubation in serum depleted medium containing 1% FBS, 1 μg/ml *Porphyromonas gingivalis* derived LPS, and 1 ng/ml IL‐1β at 37°C/5% CO_2_. LPS and IL‐1β were always freshly added to the medium. Optionally, cells were simultaneously treated with 25 μg/ml TA or 5 μg/ml PG.

#### 2.7.2 Confocal imaging

To assess cell morphology following induced inflammation for 48 h, the cells were carefully washed with PBS and fixed with 4% paraformaldehyde for 20 min at RT. Cells were stained with 5 μg/ml Alexa Fluor 568 Phalloidin and 300 nM DAPI dissolved in PBS containing 0.2% Triton X‐100 for 30 min at RT in the dark. Three non‐overlapping images were taken on two samples of each group using a Leica SP8 upright confocal laser scanning microscope with a 10×/0.40 HCPL APO CS objective.

### 2.8 Cytotoxicity

Lactate dehydrogenase (LDH) activity was measured as an indicator for membrane‐associated cell death. hGFs were incubated in 96‐well plates. Then, 100 μl of cell culture medium was collected after 24 h and mixed 1:1 with a reaction mixture according to manufacturer's instructions, and incubated at RT for 30 min. The oxidation of NADH was measured spectrophotometrically (ELx800, BioTek Instruments) at 490 nm. Results for test groups are calculated relative to a low control (C^−^, cells on tissue culture plastic [TCP]) and a high control (C^+^, cells on TCP with 1% Triton X‐100) based on Equation (1):

(Equation 1) $$\text{Cytotoxicity}\;\left(\%\right)=\frac{\;\text{Sample}-C^{-}}{C^{+}-C^{-}}\times 100\;$$

### 2.9 DNA damage

An alkaline comet assay was used to quantify permanent DNA damage in hGFs exposed to the polyphenolic molecules as described in the supplementary information. Cells in H_2_O_2_‐treated sample groups were exposed to 50 μM H_2_O_2_ for 4 h prior to sample collection. Nuclei were stained with SYBR gold at RT for 15 min in the dark shortly before imaging. Slides were imaged using a fluorescence microscope with 4× objective (Olympus IX70). At least 100 nuclei per sample were imaged and analyzed using the OpenComet plugin for ImageJ.

### 2.10 Intracellular reactive oxygen

hGFs were seeded on coated Ti coins, placed in a 48‐microwell plate, and incubated over‐night at 37°C/5% CO_2_ in complete medium to avoid any additional oxidative stress.^32^ hGFs seeded on TCP served as control. One hour before inducing inflammation, 2.5 mM N‐acetyl cysteine (NAc) or dissolved polyphenolic molecules (25 μg/ml TA; 5 μg/ml PG) were added to the cell medium. Subsequently, inflammation was induced by replacing the cell medium with 300 μl fresh medium containing LPS/IL‐1β as described above. Alternatively, 200 μM tert‐butyl hydroperoxide (TBHP) was added to the incubation medium. Following a 1 h incubation period, 2 μM CellROX DeepRed stain was added and the cells were incubated for another hour. Live/dead staining was performed with 500 nM PI for 15 min in the dark. All concentrations are given as final concentrations per well content.

To assess intracellular ROS by flow cytometry, cells were trypsinized and resuspended in PBSA. The flow cytometer (Attune NxT, Thermo Fisher Scientific) was calibrated with unstained cells, CellROX DeepRed stained TBHP treated cells, and PI‐stained Triton X‐100 permeabilized cells. At least 20,000 events were registered and gated as described in Figure S2. For all groups physical duplicates (*n*_e_ = 2) were analyzed.

### 2.11 Inflammatory response

Multianalyte profiling was performed to measure inflammatory markers secreted in cell culture medium from hGFs at 6, 24, and 48 h after induced inflammation using a Luminex 200 system. Median fluorescent intensity was analyzed using a 5‐parameter logistic line‐curve fitting method for calculating analyte concentrations in samples via the xPONENT 3.1 software (Luminex). Levels of interleukin‐6 (IL‐6), interleukin‐8 (IL‐8), monocyte chemoattractant protein‐1 (MCP‐1), and tumor necrosis factor alpha (TNF‐α) were measured using Human Cytokine/Chemokine Magnetic Bead Panel kit. The assay was performed according to the protocol provided by the manufacturer.

Total and phosphorylated (pS536) NF‐κB p65 levels in hGFs were determined 0.5 h after induced inflammation using the respective Human InstantOne™ ELISA kits (Invitrogen) according to the protocol provided by the manufacturer.

### 2.12 Statistical analysis

Data was evaluated for statistical significance using ANOVA (RStudio 1.4). Data was checked for normality using Shapiro–Wilk test and homogeneity of variances by Levene test. The ANOVA test included Ti, TA 68, TA 78, and PG 70 sample groups.

## 3 RESULTS AND DISCUSSION

### 3.1 Blood plasma and protein adsorption

Protein adsorption onto a surface determines its subsequent recognition and response by the host immune and hemostatic systems. Primarily, fibrinogen from blood plasma is found to deposit on oxide surfaces,^33^ promoting subsequent platelet adhesion. Deposited fibrinogen is then replaced by higher molecular weight proteins during prolonged exposure to blood.^34^ Therefore, it is important to investigate whether surface modifications cause a change in plasma protein adsorption on the implant surface. Further, the chemical and topographical properties of surfaces are influencing factors of the protein adsorption. Polyphenolic coatings such as investigated in this study have a roughness of approximately 11 nm, are hydrophilic and have negative zeta potential.^27^, ^35^, ^36^

**FIGURE 1:**
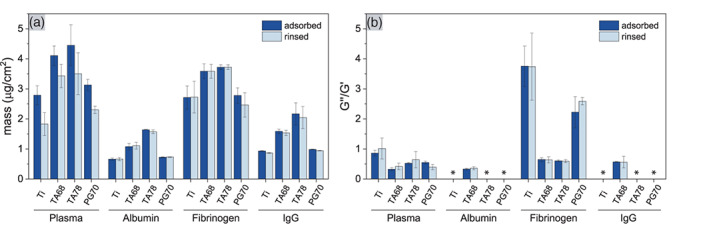
Adsorption of plasma proteins on Ti surfaces and pyrogallol (PG)‐ and tannic acid (TA)‐coated Ti surfaces as quantified by quartz crystal microbalance with dissipation monitoring (QCM‐D). (A) Adsorbed mass and (B) viscoelastic properties of citrate‐anticoagulated plasma. Plasma was diluted to 25% (v/v) in phosphate buffered saline (PBS). The amount of the three abundant plasma proteins, albumin (67 kDa), fibrinogen (340 kDa), and IgG (150 kDa), was selectively quantified at their incubation concentrations of 200 μg/ml. Polyphenolic coatings were obtained for 2 h prior to plasma protein adsorption. Four individual adsorption curves were monitored and values are given before and after rinsing with PBS (*n*_e_ = 4). (*) Rigid layers where modeling failed. Data are presented as mean ± SD

The total amount of plasma proteins absorbed on TA coatings was higher than those on PG coatings and bare Ti surfaces, as quantified by QCM‐D (Figure 1A). The protein layer formed on both TA surfaces also showed a higher viscosity and elastic modulus resulting in a more rigid protein film structure as indicated by the lower loss tangent (*G*″/*G*′) (Figures 1B and S3). Δ*D*/Δ*F* plots further attested different adsorption profiles of plasma proteins on the different surfaces (Figure S4). Together, these changes indicate a difference in the structure of the protein layer absorbed on each surface. Upon rinsing the protein layer with PBS, loosely bound proteins in the outer layers desorbed, as evidenced by a reduction in mass (Figure 1A). After the rinsing step, the viscosity of the protein layers was equal for all four studied surfaces, whereas the elastic modulus differed (Figure S3). Thus, the structural differences persisted even after desorption of the loosely attached proteins.

Due to the complexity in protein composition of human plasma, we further studied single protein adsorption of three abundant plasma proteins albumin, fibrinogen, and IgG. Albumin is the most abundant protein in plasma and governs the initial adsorption of plasma proteins on surfaces.^37^ However, albumin is later displaced by other proteins with higher affinity to the surface.^37^ Albumin formed a thin rigid layer on Ti, TA 78, and PG 70 coatings while a more dissipative structure was observed on TA 68 coatings (Figure 1B). At physiological pH, albumin adsorbs on SiO_2_ and TiO_2_ surfaces in its native conformation via electrostatic interaction and hydrogen bonding.^38^ Because the adsorption characteristics on Ti and PG 70 coatings were comparable to the adsorption on TiO_2_ (Figure S5),^39^ we assume that albumin adsorbed in its native α‐helical state.^40^ The increase in dissipation observed on TA coatings may be due to potential hydrophobic interactions and hydrogen bonding of albumin with polyphenolic molecules, leading to its denaturation.^40^ Rinsing with PBS did not cause desorption from any of the tested surfaces, suggesting that the protein layer was stable in the absence of competitive adsorption.

Fibrinogen is a key component of the coagulation cascade and its adsorption onto a surface affects the adhesion of platelets.^41^ Particularly the change in conformation and loss of its native α‐helical structure is correlated to increased platelet adhesion.^42^ Thus, we next investigated the adsorption of fibrinogen and observed a similar trend as with albumin and plasma. TA 68 and TA 78 surfaces showed an increase in fibrinogen adsorption compared to Ti and PG 70 (Figure 1A). Further, the fibrinogen layer was more rigid on TA modified surfaces (Figure 1B). Upon rinsing with PBS, the amount of fibrinogen remained unchanged, indicating a tightly bound layer. Fibrinogen showed a high binding affinity to Ti, which was even increased on TA functionalized surfaces (Figure S6). However, the splitting harmonics suggested a structural change within the formed layer on TA 68 and TA 78. This could be due to the changes in internal structure of fibrinogen. A study by Yang et al. showed that the conformation of fibrinogen upon adsorption onto polyphenolic surfaces was dependent on interactions with galloyl groups.^43^ Increasing number of galloyl groups decreased the γ‐chain activity of fibrinogen resulting in less platelet adhesion. Our results were also in good agreement with a study by Tardy et al., which showed a higher amount of fibrinogen deposition on TA/Fe^3+^ multilayers compared to albumin and plasma.^44^

Last, we studied the adsorption of the plasma protein IgG. When bound to a surface, IgG can bind C1q and activate the classical complement pathway.^45^ Similar to the other proteins studied previously, Ti and PG 70 resulted in similar IgG adsorption, whereas TA coatings resulted in a higher surface coverage of IgG (Figure 1A). While the initial IgG adsorption was more rapid on all polyphenolic coatings (Figure S7), TA 68 modified surfaces resulted in a less rigid protein layer compared to the other surfaces (Figure 1B). Splitting harmonics suggested a change in internal layer structure especially on TA 68 coatings (Figure S7). In practice, a low adsorption of IgG may be beneficial since in vivo studies have shown that IgG‐modified implants recruited more leukocytes.^46^, ^47^ Thereby, the inflammatory response caused by the IgG‐modified Ti implants delayed the early tissue integration of the modified implants.

Although we observed a clear difference in blood plasma adsorption onto the different polyphenol‐coated surfaces, the impact on the level of individual proteins remains complex. The potential inhibition of protein rearrangement may further impact the adsorption and conformation of complement and coagulation components.

### 3.2 Interactions with human whole blood

To evaluate whether polyphenolic coatings can modulate inflammatory or thrombotic responses after the implant placement, we explored the interactions of whole blood with our surface coatings. Since polyphenols, such as TA, interact with blood components,^48^ we focused on the activation of the complement and coagulation system. These two systems largely determine whether the surface is accepted by the host upon implantation.^49^

#### 3.2.1 Complement activation

**FIGURE 2:**
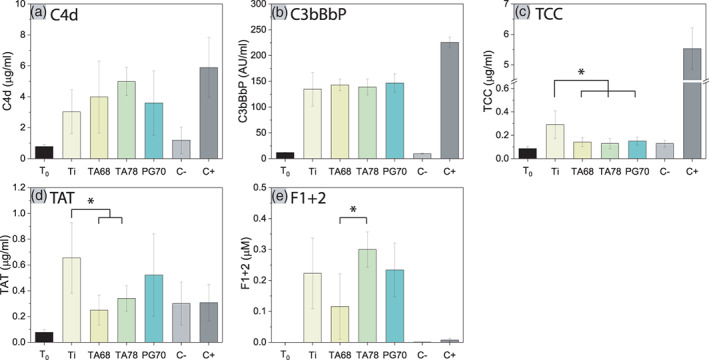
Interaction of different surface coatings with human whole blood. Markers of (A–C) complement activation and (D, E) coagulation activation after 30 min incubation of human whole blood with Ti, pyrogallol (PG)‐, and tannic acid (TA)‐coated surfaces at 37°C. T 0 refers to the blood sample before incubation and serves as baseline levels. C− and C+ are negative and positive controls with blood without any activator and blood incubated with *Escherichia coli*. Blood was taken from three donors and three samples per group were analyzed (*n*_e_ = 9). Values represent mean ± SD. (*) *p* value < .05

The complement system is one of the key components of innate immunity and can be activated via three pathways: the classical, the lectin, and the alternative pathway.^50^ Along the activation cascade, different activation products are generated, which in turn play pleiotropic functions in immune responses.^51^ Many of these products can be used as activation markers. C4d is an activation product common for both the classical and the lectin pathway.^50^ It was detected at elevated levels on all surfaces, implying the activation of either or both of these pathways (Figure 2A). C3bBbP is the C3‐convertase of the alternative pathway of the complement system.^50^ The formation of C3bBbP complex was found in blood samples incubated with all surfaces, suggesting the activation of the alternative pathway (Figure 2B). This is in agreement with known activation of the alternative pathway on surfaces bearing nucleophiles, such as polyphenolic hydroxyl groups, and on protein layer formed on surfaces.^52^, ^53^ Regardless of the activation, all pathways of the complement system converge at the central C3 and C5 proteins, and subsequently combine with C6–C9 to form termination complement complex or TCC.^50^ Although elevated levels of C4d and C3bBbP were detected for all surfaces compared to negative control (C−), polyphenolic coatings did not induce increased levels of TCC (Figure 2C). While Ti surfaces yielded low TCC levels compared to the positive control, TA and PG coatings maintained TCC concentrations on the level of C−. These results suggest that the modified surfaces may inhibit the further amplification process of the complement cascade and reduce the release of C3a and C5a.

#### 3.2.2 Coagulation activation

The coagulation system has a critical role in hemostasis.^54^ It can be activated through two pathways, intrinsic and extrinsic, generating functional activation products along these two cascades.^55^ Ti surfaces induced high levels of both TAT and F1 + 2 (Figure 2D,E). As products from thrombin activation, they mark the activation of coagulation. This is in accordance with a previous study showing the induction of blood coagulation by Ti surfaces.^56^ Both TA 68 and TA 78 modified surfaces had lower TAT levels, but TA 78 induced an increase in F1 + 2 (Figure 2D,E). TAT and F1 + 2 levels for PG surfaces were comparable to Ti surfaces within the margin of error. These results confirm a previous study where low amount of TA did not affect the coagulation of blood.^57^ Extrinsic activation of the coagulation system by implant surfaces is known to be dependent on surface charge and hydrophobicity.^58^, ^59^ Our results showed that the coated surfaces maintain the thrombogenic activities of bare Ti surfaces, despite their altered surface chemistry. This may also preserve the osseointegrative property of Ti, which is linked to a thrombin stimulated osteoblast proliferation.^60^

#### 3.2.3 Platelet activation

**FIGURE 3:**
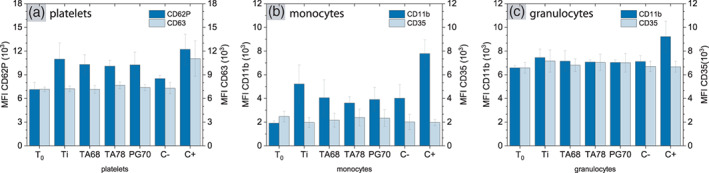
Activation of platelets, monocytes, and granulocytes. Blood was incubated with Ti, pyrogallol (PG)‐, and tannic acid (TA)‐coated surfaces for 30 min. (A) The activation of platelets was quantified by the expression levels of CD62P and CD63 using flow cytometry. The activation of (B) monocytes and (C) granulocytes was measured by the levels of CD11b and CD35. Experiments were conducted in triplicate with blood from three donors each (*n*_e_ = 9). Results are presented as median fluorescent intensity (MFI) ± SD. No statistical significance was obtained

Platelets play a vital role in hemostasis and inflammation.^61^ Their activation is closely connected to both the complement and the coagulation system.^62^ To evaluate whether different surface coatings elicit platelet activation, we measured the expression of two surface activation markers, CD62P and CD63, using flow cytometry. Upon activation, CD62P (P‐selectin) rapidly mobilize from α‐granules to the platelet surface, by which it can mediate the binding of platelets to cells expressing P‐selectin glycoprotein ligand‐1 (PSGL‐1).^63^ Expression levels of CD62P increased after incubation with all surfaces, implying platelet activation (Figure 3A). In contrast, CD63 is not associated with platelet adhesion, but mediates platelet spreading and aggregation. CD63 expression remained as low as observed in both T_0_ and C‐, suggesting that different coatings did not induce a significant change in platelet morphology (Figure 3A). CD62P and CD63 expression together indicated that all different surfaces induced an early activation of platelets, but not a significant change in their aggregation

#### 3.2.4 Monocyte and granulocyte activation

Activation of monocytes and granulocytes is a hallmark for acute inflammation. Both activation markers CD11b (macrophage‐1 antigen) and CD35 (complement receptor 1) remained unchanged at the background level observed in the negative control (Figure 3B,C). The expression of both CD11b and CD35 is closely linked to the activation of the complement and coagulation systems.^51^ Combined with the results described earlier (Figure 2), these results imply that although different surface coatings induced the activation of these two systems, the activation was not sufficient to elicit significant cellular responses against these surfaces.

#### 3.2.5 Cytokine profile

**FIGURE 4:**
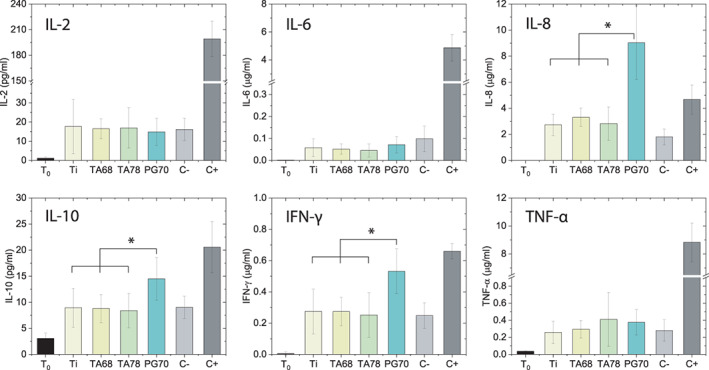
Expression profile of selected plasma cytokines. Plasma cytokines were measured after 4 h incubation of blood with Ti, tannic acid (TA),‐ and pyrogallol (PG)‐modified surfaces. Experiments were conducted in triplicates with blood from three donors each (*n*_e_ = 9). Results are presented as mean ± SD. Data for other cytokines are presented in Figure S8. (*) *p* value < .05

A panel of 27 plasma cytokines was analyzed after incubation with different coating surfaces. Six cytokines most relevant for inflammation are presented in Figure 4. In general, TA‐coated surfaces did not induce a significant change in expression of any cytokines while PG‐coated upregulated leukocyte chemotactic cytokines (Figures 4 and S8). Upregulation of IL‐8, a vital chemoattractant for leukocyte recruitment,^64^ has previously been found after incubation of neutrophils with penta‐galloyl glucose (PGG).^65^ Thereby, the wound healing process may be supported by reducing the pro‐inflammatory reaction of neutrophils.

### 3.3 Antioxidant capacity

**FIGURE 5:**
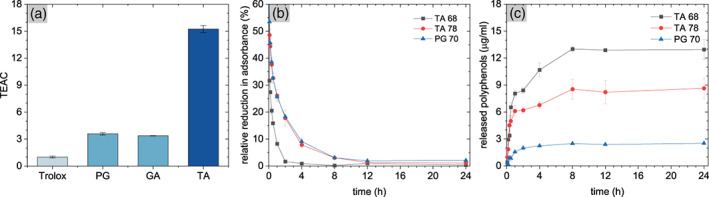
Antioxidant capacity of tannic acid (TA)‐ and pyrogallol (PG)‐coated surfaces. (A) Trolox equivalent antioxidant capacity (TEAC) of polyphenolic molecules dissolved in water measured after 6 min (*n*_e_ = 3). (B) Absorbance reduction of 2,2′‐azino‐bis(3‐ethylbenzothiazoline‐6‐sulfonic acid (ABTS) in presence of coated coins. (C) Release of polyphenolic molecules from one coated coin per 1 ml water. Results are presented as mean ± SD

Dissolved polyphenols are generally considered as antioxidant molecules with a structure‐dependent capacity to scavenge free radicals.^13^ Thus, we investigated the antioxidant capacity of our molecules and whether the antioxidant properties of TA and PG are conserved when deposited on Ti surfaces. As shown in Figure 5A, PG and gallic acid (GA) showed a 4‐times higher antioxidant capacity compared to trolox. GA is the structural subunit of TA, which can consist of up to 10 GA units. TA reached a TEAC value of 16 (Figure 5A). This corresponds to four GA units and may suggest that either only the outer GA groups in TA are active in scavenging radicals, or that commercial TA has a high degree of fragmentation. The latter assumption is supported by the evidence of a high GA content in commercial TA.^66^ As the radical formation of polyphenols is pH dependent,^66^ we observed a peak in TEAC for TA at pH = 7 before a reduction in TEAC occurred with increasing pH (Figure S9A). This behavior was not observed for PG and can be explained by the onset of oxidation for TA at pH = 7, whereas PG oxidizes already at lower pH (Figure S10). Upon oxidation with NaMnO_4_, the TEAC of TA showed the same behavior, indicating the impact of the oxidation state of the molecule on its radical scavenging capacity (Figure S9B). In the subsequent analysis of the TEAC of TA and PG coatings, we observed that modified surfaces rapidly discolored ABTS solutions (Figure 5B). To investigate whether this was an effect of released or surface‐confined molecules, we quantified the amount of polyphenolic molecules released from the surface. TA 68 coatings showed the highest amount of released molecules with 13 μg/ml, which corresponds to 7.6 μM TA, while TA 78 and PG 70 surfaces released only 8.5 μg/ml (5 μM TA) and 2.5 μg/ml (20 μM PG) (Figure 5C). This can be attributed to the larger thickness of TA 68 coatings compared to TA 78 and PG 70 coatings.^27^, ^28^ However, the full reduction of the ABTS solution by the coatings indicated a calculated concentration of more than 10 μM (17 μg/ml) of TA and 30 μM (4 μg/ml) of PG. Thus, not only the released molecules were responsible for the observed antioxidant effect, but also the active surfaces. The released amount of TA molecules further confirms that the concentration is below the threshold where adverse effects with blood were observed.^48^

TA coatings disassemble in strong acidic and alkaline condition.^27^ Since inflammation can cause local acidosis,^67^ we further investigated the release of molecules at various pH levels. The dissolution kinetics of TA and PG coatings confirmed an initial burst release of molecules as shown in Figure 5C, which tapered off after 4 h (Figure S11A). The least reduction of the polyphenolic layer mass was observed at neutral pH (Table S1). Either increasing or decreasing the pH resulted in a higher release of the layers. At pH = 3, TA coatings rapidly dissolved within 2 h whereas PG coatings remained more stable (Figure S11B).

Cell culture medium is an additional factor influencing the release of polyphenols in cell experiments. We attempted to quantify the release of polyphenols in DMEM, but simultaneous protein adsorption onto TA and PG coated surfaces made it unfeasible (Figure S12). Although the release in medium could not be directly measured, we expect that some molecules are being released to provide an antioxidant effect.

### 3.4 Cytotoxic effect on hGFs

Ti is a remarkable material for bone implants.^68^ However, improvement of soft tissue integration is often neglected. Soft tissue integration plays an important role for dental implants, where fibroblasts are responsible for remodeling granular tissue and provide a close seal of the gingiva to protect the implant surface from the oral environment.^69^ Hence, we proceeded to explore whether PG and TA coatings affect the response of hGFs under inflammatory conditions.

**FIGURE 6:**
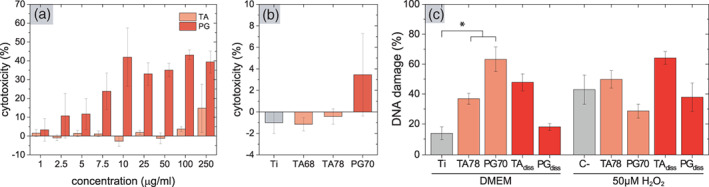
Cytotoxicity of tannic acid (TA) and pyrogallol (PG) on human gingival fibroblasts (hGFs). Cytotoxicity of (A) dissolved polyphenols (*n*_e_ = 6) and (B) polyphenolic‐coated surfaces on hGFs as quantified by lactate dehydrogenase (LDH) assay (*n*_e_ = 3). (C) DNA damage of hGFs in presence of dissolved polyphenolic molecules (100 μg/ml TA, 5 μg/ml PG) or polyphenol‐coated surfaces (*n*_e_ = 6). hGFs were cultured in complete medium or in the presence of H_2_O_2_. Note that the relative concentration of released TA and PG from coins (Figure 5C) into the medium (panel B) is potentially higher than direct supplementation (panel A) due to the working volume of 200 μl per well. Results are presented as mean ± SD. (*) *p* value < .05

LDH assay showed low level of cytotoxicity towards hGFs for TA concentrations up to 250 μg/ml, whereas PG concentrations above 5 μg/ml showed considerable cytotoxic effect (Figure 6A). The higher apparent cytotoxic effect can be explained by the 10 times higher molar concentration of PG compared to TA. hGFs seeded on either TA or PG coatings showed low cytotoxicity (Figure 6B), which was consistent with the low non‐toxic concentrations of TA and PG released from the coatings (Figure 5C). Furthermore, cells spread and adopted the typical spindle‐shaped cell morphology of healthy fibroblasts on all tested surfaces (Figure S13), which correlates with the obtained LDH results.

These results show that the coatings did not influence the integrity of hGFs cell membrane. However, polyphenolic compounds and their oxidation products, quinones, are potentially mutagenic agents.^70^ In addition to their antioxidant effect, polyphenols may also elicit pro‐oxidant properties, such as the production of ROS, which may lead to DNA damage and apoptosis.^71^, ^72^, ^73^ Indeed, both TA 78 and PG 70 coatings showed higher DNA damage compared to bare Ti surfaces (Figure 6C). While the DNA damage caused by dissolved TA was slightly higher compared to the TA coating, dissolved PG did not induce more DNA damage than Ti. The high DNA damage caused by coatings could be an effect of the concentration of the released molecules in the working volume of the assay but cannot be correlated with cytotoxic effects (Figure 6A). Interestingly, both dissolved PG and PG coatings reduced the DNA damage induced by 50 μM H_2_O_2_ compared to the negative control (TCP). In contrast, TA caused higher DNA damage in combination with H_2_O_2_ (Figure 6C).

The mutagenicity and toxicity of polyphenols can occur through either coupling of the highly reactive phenolic compounds to cell components (arylation) or redox reactions creating reactive radical species.^74^ Once polyphenolic compounds are oxidized to quinones, mutagenic effects have been shown to occur through electrophilic coupling reactions.^75^ This pro‐oxidant effect has been observed for GA in the absence of H_2_O_2_.^72^ Since TA is a hydrolysable molecule, its degradation can yield GA. Such degradation processes have been observed in DMEM, where the resulting GA formed ROS and inhibited cell growth.^76^ This means that in vitro studies are highly dependent on the culture conditions and the concentration of the polyphenols.

### 3.5 Intracellular reactive oxygen species

**FIGURE 7:**
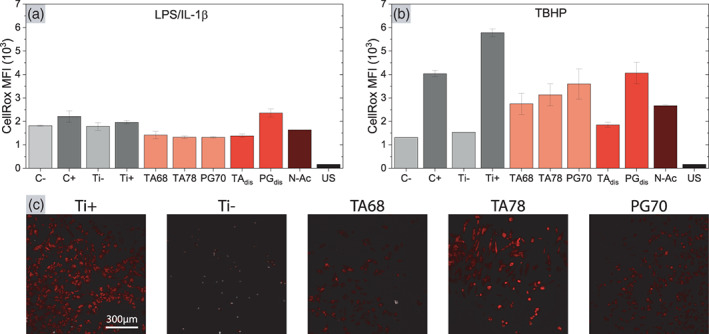
Intracellular reactive oxygen species (ROS) level. (A) CellROX intensity in human gingival fibroblasts (hGFs) after (bacteria derived) lipopolysaccharide (LPS)/IL‐1β induced inflammation as determined by flow cytometry. (B) Intracellular ROS levels in hGFs after exposure to 200 μM tert‐butyl hydroperoxide (TBHP). In both experiments, hGFs were inflamed for 2 h. CellROX DeepRed was added after 1 h. Concentrations for dissolved polyphenols were 250 μg/ml TA and 5 μg/ml pyrogallol (PG). 10 k cells were registered for two individual runs (*n*_e_ = 2) and results are presented as mean ± SD. (C) Representative images show the intracellular ROS in hGFs inflamed by TBHP

Cells can be subject to oxidative stress caused by the release of reactive oxygen species (ROS) during inflammation. First, we investigated whether the inflammatory response of hGFs induced by LPS/IL‐1β is connected to intracellular ROS. CellROX staining showed no detectable level of intracellular ROS in hGFs (Figure 7A). The inflamed positive controls on TCP (C+) and Ti coin (Ti+) showed almost the same signal as the negative controls C− and Ti− that were not exposed to LPS/IL‐1β. All groups showed an unspecific CellROX signal intensity compared to the unstained control (US). Although ROS is often associated with phagocytes,^77^ exposing fibroblasts to *P. gingivalis*‐derived LPS and IL‐1 has also resulted in elevated mitochondrial ROS expression.^78^, ^79^ Our negative results could be an effect of the specific primary cells we used in this study. We observed that our hGFs did not show an inflammatory response after LPS stimulation (Figure S14), and under certain circumstances, hGFs exposed to LPS can respond with LPS tolerance.^80^

Besides endogenous ROS production, oxidative stress in hGFs can also be induced externally. During inflammation, recruited granulocytes and macrophages produce ROS, such as H_2_O_2_, to remove foreign objects by phagocytosis.^81^ ROS, which are able to cross cell membranes,^82^ can then induce oxidative stress and apoptosis in the surrounding cells.^83^ Upon exposing hGFs to TBHP, dissolved TA reduced the intracellular ROS level close to that of the negative control (Figure 7B,C), demonstrating the high antioxidant capacity of TA. Despite its lower concentration, TA outperformed the higher concentrated antioxidant N‐acetyl cysteine in reducing intracellular ROS upon TBHP stimulation. Dissolved PG also reduced the ROS level, which is in correlation with its TEAC (Figure 5A). Importantly, we also observed a reduction of intracellular ROS levels for hGFs cultured on coated TA and PG coins. Of the tested surfaces, TA 68 was found to be most effective in reducing intracellular ROS, probably due to the higher number of molecules being released from the surface (Figure 5C) or the non‐oxidized nature of the molecule. Both possibilities are plausible and in accordance with literature as well as our own results attesting decreased radical scavenging capacity of oxidized polyphenols (Figure S9).^84^ None of the groups showed cytotoxic effects during TBHP treatment as determined by PI staining (Figure S15), which supports our LDH assays (Figure 6B).

In comparison to our DNA‐damage results, which indicated that polyphenols can be pro‐oxidant, ROS determination by flow cytometry did not indicate redox cycling in the presence of TBHP (Figure 7B). Correlation of DNA damage and intracellular reactive oxygen thus needs further research. However, the experiment verified that TA and PG coatings maintain their antioxidant properties in DMEM, despite protein adsorption on the coating surface (Figure S12). Thus, potential reaction of polyphenols with proteins through addition‐reactions did not inhibit the radical scavenging properties of the molecules, which agrees with previous studies.^85^, ^86^

### 3.6 Cytokine expression and regulatory effect of polyphenols

**FIGURE 8:**
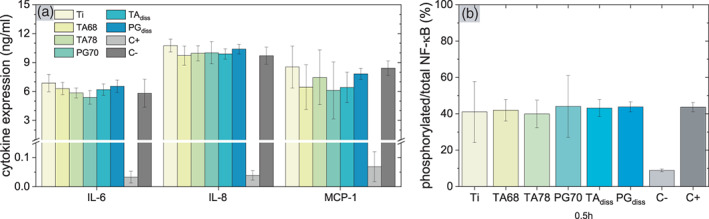
Cytokine expression and For Peer Review NF‐κB activation in human gingival fibroblasts (hGFs). (A) Multiplex analysis of inflammatory cytokine expression in hGFs 24 h after inflammation with (bacteria derived) lipopolysaccharide (LPS) and IL‐1β (*n*_e_ = 6). (B) Ratio of phosphorylated to total NF‐κB p65 in hGFs 0.5 h after inflammation by LPS and IL‐1β (*n*_e_ = 3). Concentrations for dissolved polyphenols were 250 μg/ml TA and 5 μg/ml pyrogallol (PG). Individual values for total and phosphorylated NF‐κB p65 are given in Figure S16D. Results are presented as mean ± SD. No statistical significance was obtained

After detecting no ROS in LPS/IL‐1β stimulated hGFs, we continued to assess the expression of inflammatory cytokines. On Ti surfaces hGFs showed high levels of IL‐6, IL‐8, and MCP‐1 (Figure 8A). This is in good relation with reported response of hGFs to IL‐1β and LPS derived from *P. gingivalis*.^87^, ^88^ TNF‐α levels were below the detection limit and thus not shown. Neither dissolved polyphenols nor the modified surfaces affected the inflammatory response 24 h after stimulation with LPS/IL‐1β (Figure 8A). Similar observation was made 6 and 48 h after inducing inflammation (Figure S16).

Contrary to our results, other studies have found that TA and PG reduced the expression of pro‐inflammatory cytokines in various human cells stimulated by LPS.^89^, ^90^, ^91^ Further, several other polyphenolic molecules have been shown to reduce the inflammatory response in hGFs after stimulation with LPS.^18^, ^92^, ^93^ However, in our study, the inflammatory trigger was rather IL‐1β than LPS, as no inflammatory stimulus was observed for LPS alone (Figure S14). Under stimulation with IL‐1β, polyphenols may not be able to curb the inflammatory cell response, as also suggested by a study using quercetin coatings.^94^

Since the expression of the pro‐inflammatory cytokines upon exposure to LPS and IL‐1β has been associated with NF‐κB signaling pathways,^95^, ^96^ we investigated whether TA and PG affect NF‐κB signal transduction. We found equal level of phosphorylation of NF‐κB p65 in hGFs for all tested samples 30 min after induction of inflammation (Figure 8B). Contrary to previous studies,^17^, ^92^, ^95^, ^96^, ^97^ dissolved TA and PG did not reduce NF‐κB phosphorylation, which clearly correlated with the cytokine expression. We assume that our coatings could not inhibit the inflammatory response either due to the hGFs we used, or due to a low concentration of released polyphenols. Furthermore, other inflammatory signaling pathways besides NF‐κB p65 signaling should be considered, such as P13‐K, MAPK, ERK, and p53.^98^, ^99^ Thus, further studies need to be conducted to reliably discern the effect of the simultaneous induction of inflammation by LPS and IL‐1β and to investigate the dose‐dependent effect of polyphenolic treatments in hGFs.

### 3.7 Significance of the results on early wound healing processes

Protein adsorption is a key factor in the foreign body response. Polyphenolic coatings did not significantly change the amount of adsorbed proteins. Although the coatings activated the coagulation and complement system, we did observe statistically significant reduced levels of TCC. This may indicate a lower inflammatory response compared to bare Ti surfaces. Further, no upregulation in pro‐inflammatory cytokines confirmed that our coatings did not stimulate inflammation. Regarding the anti‐inflammatory effect, we observed reduced levels of intracellular ROS in hGFs cultured on coated surfaces. These results suggest that nonspecific oxidative stress caused by immune cells towards other cells can be reduced during the early wound healing process. However, inflammation in hGFs induced by LPS/IL‐1β could not be curbed. These cells were chosen to particularly study the healing of soft tissue around dental implants, as they are the first cells to arrive at the wound site after blood cells to remodel the blood clot.^69^ In addition to hGFs, future research should evaluate the effect of polyphenolic coatings on other cell types, particularly immune cells such as macrophages,^100^ before a more general conclusion can be drawn. Regarding other types of Ti implants, the respective cell types that are in primary contact with the implant need to be studied as well. Together, these experiments will then lay the foundation for further research that addresses the complex foreign body response in appropriate in vivo models.

## 4 CONCLUSIONS

In this study, we investigated the effect of TA and PG coatings on the early wound healing processes and cell response under inflammatory conditions. The polyphenolic surface modifications altered the initial blood protein layer formed on the surfaces, which is a key factor in the subsequent complement, coagulation, and platelet activation as well as cell adhesion. TA coatings generally showed higher protein adsorption compared to PG and Ti. Further, TA and PG coatings activated the classical, lectin, and alternative complement pathways like Ti surfaces. However, the formation of the terminal complement complex was reduced on the TA and PG coated surfaces. The thrombogenic properties of Ti were retained and high levels of TAT and F1 + 2 were found for TA and PG coated surfaces. This was corroborated by platelet activation. In contrast, monocytes and granulocytes were not activated by Ti or polyphenolic coatings, which was represented in the cytokine expression.

The modified surfaces showed antioxidant properties, which reduced intracellular reactive oxygen levels in hGFs. While the coatings showed good cytocompatibility, potential increase in DNA damage was observed. Upon inflammation of hGFs with LPS/IL‐1β, polyphenolic coatings were not able to reduce the expression of pro‐inflammatory cytokines. This was linked to the activation of the NF‐κB p65 signaling pathway. Since inflammatory responses and signaling pathways are complex cell specific processes, further studies are needed to evaluate the ability of polyphenolic surface modifications to support early wound healing processes.

## CONFLICT OF INTEREST

The authors declare no conflict of interest.

## Supporting information


**Appendix S1:** Supporting information

## Data Availability

Data available on request from the authors.
