# Supplementary material for: Characterization of the foreign body response of titanium implants modified with polyphenolic coatings
Source: J Biomed Mater Res A. 2022 Feb 26;110(7):1341–55. doi: 10.1002/jbm.a.37377 (PMC9305744; doi:10.1002/jbm.a.37377)
Supplement: Supplementary file 1 — Appendix S1: Supporting information [file JBM-110-1341-s001.docx]

*Supplementary Information*

Characterization of the Foreign Body Response of Titanium Implants Modified with Polyphenolic Coatings

Florian Weber^a^, Huy Quang Quach^b^, Mathias Reiersen^a^, Sadaf Yosef Sarraj^a^, Dyala Nidal Bakir^a^, Victor Aleksander Jankowski^a^, Per H. Nilsson^b,c^, Hanna Tiainen^a*^

^a^Department of Biomaterials, Institute of Clinical Dentistry, University of Oslo, Norway

^b^Department of Immunology, Institute of Clinical Medicine, University of Oslo, Norway

^c^Department of Chemistry and Biomedical Sciences, Linnaeus University, Kalmar, Sweden

Table of Contents

[Abbreviations S1](#_Toc64285037)

[Supplementary methodological considerations S2](#_Toc64285038)

[Supplementary figures S4](#_Toc64285039)

[References S17](#_Toc64285040)

# Abbreviations

Tannic acid (TA), gallic acid (GA), pyrogallol (PG), tannic acid coatings obtained at pH = X.Y (TA XY), PG coatings obtained at pH = 7.0 (PG 70), trolox equivalent antioxidant capacity (TEAC), tissue culture plastic (TCP).

# Supplementary methodological considerations

**Chemicals.** Tannic acid (TA, LOT#MKBN9606V), pyrogallol (PG, LOT#MKBS3610V), HEPES (BioPerformance, ≥ 99.5%), sodium metasilicate pentahydrate (Si_aq_, ≥ 95%), ABTS (≥ 98%), K_2_(SO_4_)_2_ (puriss, ≥ 99.0%), NaMnO_4_⋅H_2_O (≥95%), FeCl_3_⋅6 H_2_O, K_4_Fe(CN)_6_⋅3 H_2_O (ReagentPlus, ≥ 98.5%), N-acetyl cysteine (NAc, ≥99%), hydrogen peroxide (H_2_O_2_, 30%), and tert-butyl hydroperoxide (TBHP, 70% in H_2_O) were purchased from Sigma Aldrich. NaCl and NaOH were supplied by VWR at ACS grade. Fixative-free lysis buffer for blood experiments was purchased from Invitrogen^TM^ (Cat# HYL250).

Human citrated plasma, fibrinogen (50−70%), fatty acid free bovine serum albumin (≥96%), and IgG from bovine serum (≥95%) were purchased from Sigma Aldrich.

**hGF cell culture.** Human gingival fibroblasts (hGF) were purchased from Provitro. DMEM was supplied by Sigma Aldrich and GlutaMAX was purchased from Gibco. Recombinant human IL-1β and *Porphyromonas gingivalis*-derived lipopolysaccharide (LPS) were obtained from Sigma Aldrich and InvivoGen, respectively.

**hOB cell culture.** Human osteoblasts (hOB, Lonza) were cultured in osteoblast basal medium supplemented with OGM-SingleQuots (Lonza), containing 10% fetal bovine serum (FBS), 0.1% amphotericin/gentamicin, and 145 μM ascorbic acid. Inflammation was induced in osteoblast growth medium containing 10% FBS, 1 μg/ml *Porphyromonas gingivalis* derived LPS and 1 ng/ml IL-1β.

**Analytical kits.** Molecular probes were supplied as follows. CD42a-FITC (BD Biosciences, San Jose, CA), CD63-PE-Cy7 (Invitrogen, Carlsbad, CA), CD62P-PE (BD Biosciences), CD45-Pacific Orange (Introvigen^TM^), CD14-PerCP (BD Biosciences), CD11b-APC/Fire 750 (Biolegend, San Diego, CA), and CD35-Alexa Fluor 647 (Introvigen^TM^).

Cellular stains Alexa Fluor 568 Phalloidin, DAPI, CYBR gold, and CellROX Deep RED were supplied by Thermo Fisher Scientific. Propidium iodide (PI) was purchased from Sigma Aldrich.

ELISA kits for blood experiments were supplied as follows. TAT and F1+2 ELISA kits were from Siemens Healthcare Diagnostic Products GmbH, C4d (COMPL C4d RUO, Svar Live Science AB), and TCC (COMPL TCC RUO, Svar Live Science AB). C3bBbP levels were analysed according to an assay developed in house.^1^

Cytokine kit Bio-Plex Pro (Bio-Rad Laboratories, CA, USA) used for blood experiments included the following panel of 27 cytokines and chemokines. FGF basic, Eotaxin, G-CSF, GM-CSF, IFN-γ, IL-1β, IL-1ra, IL-2, IL-4, IL-5, IL-6, IL-7, IL-8, IL-9, IL-10, IL-12 (p70), IL-13, IL-15, IL-17A, IP-10, MCP-1 or MCAF, MIP-1α, MIP-1β, PDGF, RANTES, TNF-α, VEGF.

Multianalyte profiling for hGFs was conducted with a Human Cytokine/Chemokine Magnetic Bead Panel Kit (EMD Millipore, Billerica, MA), which included IL-6, IL-8, MCP-1, and TNF-α. Total and phosphorylated (pS536) NF-κB p65 levels were determined using Human InstantOne™ ELISA kits (Invitrogen).

Reaction of hGFs and hOBs towards LPS derived from *P. gingivalis* and IL-1β was analysed with an IL-6 human ELISA kit (Invitrogen, Catalog # KHC0061).

Cytotoxicity was determined using an LDH kit provided by Roche Diagnostics, Mannheim, Germany.

**Polyphenol oxidation.** The oxidation of TA and PG was monitored using a UV-Vis spectrophotometer (Lambda 25, PerkinElmer). Polyphenols were dissolved at a concentration of 1 mg/ml in 10 mM phosphate buffer containing 150 mM NaCl at pH = 7.0. The absorption was quantified from 190 nm to 1000 nm at a slit-width of 1 nm using a quartz cuvette with 10 mm path length.

**Comet assay.** An alkaline comet assay was used to quantify permanent DNA damage in hGF exposed to the polyphenolic molecules. Either coated surfaces were placed in well of a 96-well plate and cells were seeded on them, or cells were seeded directly on the TCP and treated with 100 μg/ml TA (TA_diss_) or 5 μg/ml PG (PG_diss_). The cell density was 3.5 × 10^3^ cells per well. After seeding, hGFs were allowed to adhere for 2 h. Cells in H_2_O_2_-treated sample groups were exposed to 50 µM H_2_O_2_ for 4 h prior to sample collection. Cells were detached from sample discs or wells after 24 h with trypsin/EDTA, re-suspended in 225 µl culture media and immediately put on ice. After centrifugation (5 min at 200 g), the supernatant was removed and the cell pellet was mixed in 26 µl low melting point agarose. The agarose-cell suspension was divided into two equal drops of 12 µl and placed on an agarose coated microscope slide. Slides were stored at 4^○^C for 10 min for complete gelation. A high control (cells on TCP) was placed in a slide holder filled with cold 3% H_2_O_2_ for 5 min. All slides were then placed in slide holders filled with a cold lysis solution (2.5 M NaCl, 0.1 M EDTA, 10 mM Tris-HCl, pH = 10, 1% triton X-100) and stored at 4^○^C for 1 h. All slides were placed in alkaline electrophoresis solution (0.3 M NaOH, 1 mM EDTA) for 20 min at 4°C for DNA unwinding before electrophoresis was run for 30 min at 25 V and 350 mA. Slides were neutralized with cold PBS and d-H_2_O for 10 min each and dried overnight at RT.

Nuclei were stained with SYBR gold at RT for 15 min in the dark shortly before imaging. Slides were imaged using a fluorescence microscope with 4× objective (Olympus IX70, Olympus, Tokyo. Japan). At least 50 nuclei per droplet (100 per sample) were imaged and analyzed using the OpenComet plugin for ImageJ. DNA damage was calculated using %DNA-in-tail according to following equation.

$$DNA damage \left( \% \right)=\frac{sample-negative control}{positive control-negative control}*100$$

**Voight viscoelastic modelling.** QCM-D raw data was imported in DFind (V.1.2.7, BiolinScientific) and periods were set accordingly to model the protein adsorption. *SmartFit* models with frequency dependent viscosity and elastic modulus were obtained based on the 3^rd^, 5^th^, 7^th^, and 9^th^ harmonic. Period analysis was conducted by averaging the signal for 60 s at the end of each period with an offset of 20 s.

The computed viscoelastic parameters for the elastic modulus (*μ*) and viscosity (*η*) can be expressed in the complex shear modulus (*G*) according to the following equation.^2^

$$G=G^{'}+iG^{''}=\mu+i2\pi\eta f$$

The loss tangent (*G*’’/*G*’) is an indicator of the viscoelastic properties. For *G*’’/*G*’ < 1, adsorbed layers behave solid-like, whereas for values above a more liquid-like layer is formed.

# Supplementary figures


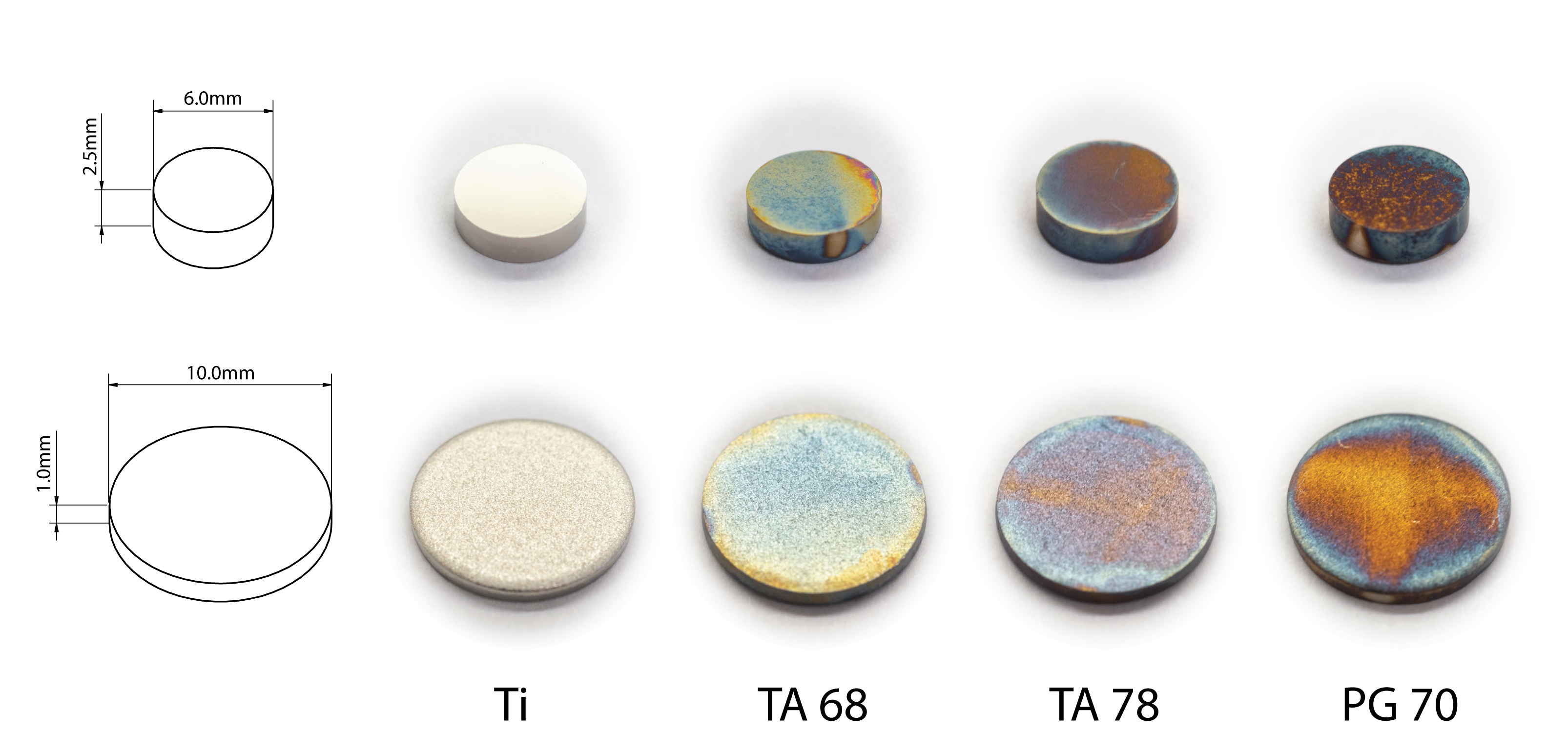


**Figure S1:** Titanium (Ti) surfaces used in this study before and after coatings with TA at pH = 6.8 and pH = 7.8, and PG at pH = 7.0 for 24 h. Small coins were used throughout the study besides in the flow cytometry experiments in which intracellular ROS were quantified. In later experiments, larger coins were used to increase the cell number per sample. The available surface area for polyphenol release for the small coins is 104 mm^2^. Large coins have a surface area of 220 mm^2^.


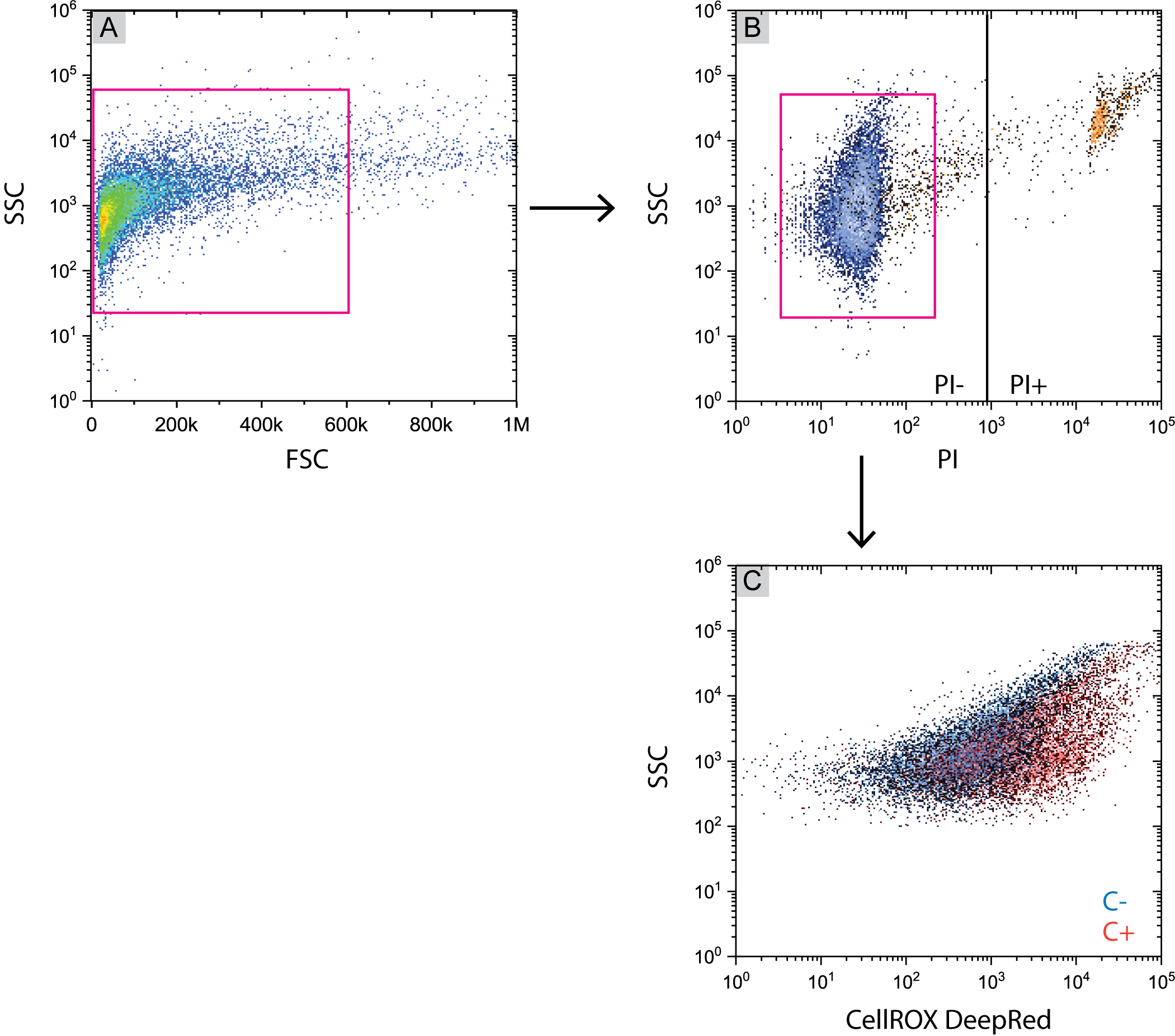


**Figure S2:** Gating strategy for detecting intracellular reactive oxygen species using CellROX DeepRed staining. (A) hGF population was gated based on side scatter area (SSC) and forward scatter area (FSC). (B) Then, the live cell population (PI-) was chosen based on the propidium iodide staining to quantify the mean CellROX intensity (C).


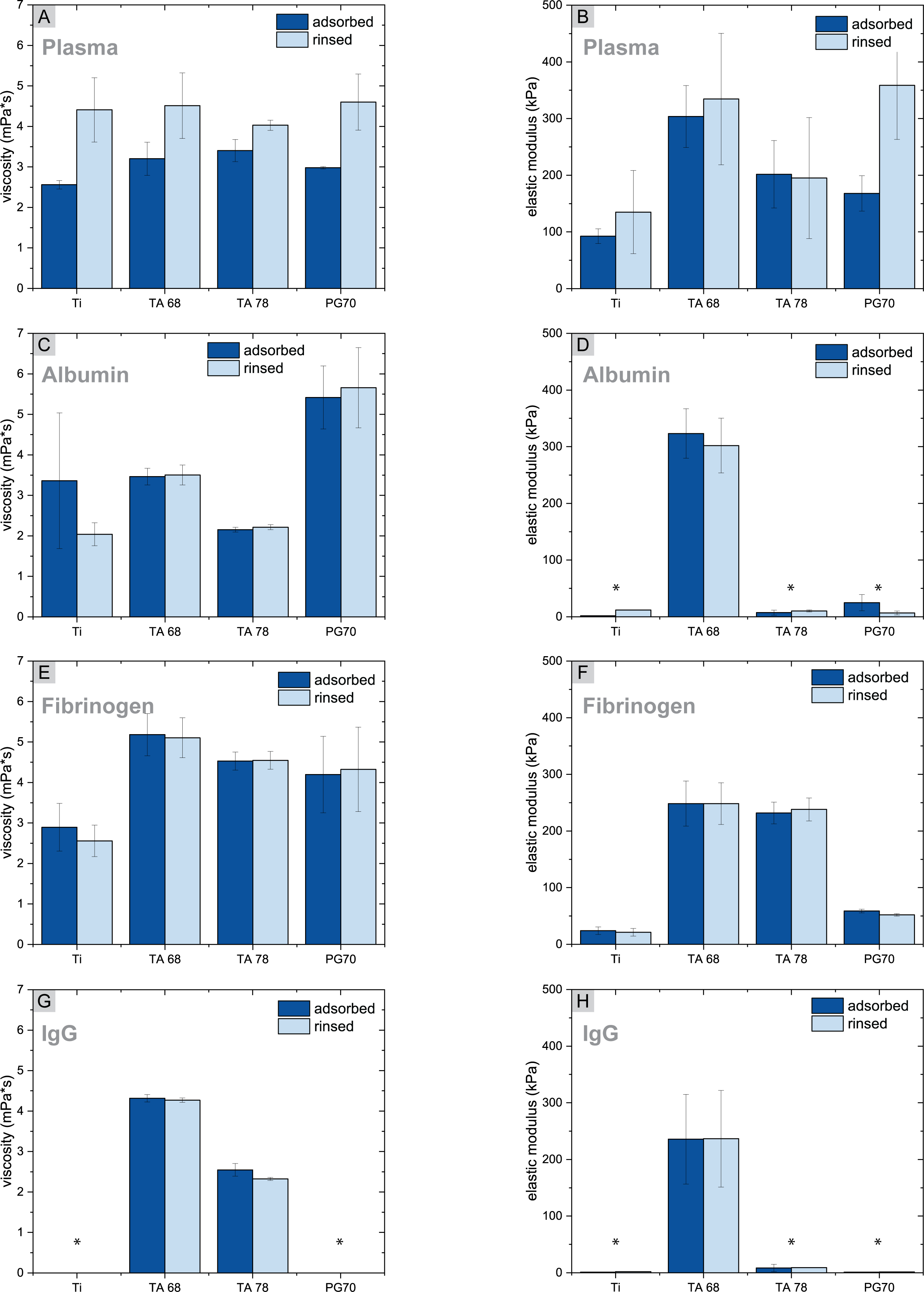


**Figure S3:** Viscoelastic properties of proteins pellicle formed by the adsorption of (A, B) human plasma, (C, D) bovine serum albumin, (E, F) fibrinogen, and (G, H) immune globulin G. Viscosity and elastic modulus of the protein layer were modeled using DFind after the adsorption and a rinsing step. Values are shown as mean ± SD (n_e_ = 4). PG and TA coatings were formed on the Ti sensor for 2 h prior to the adsorption of proteins. These modeled parameter are taken with caution as the applied *SmartFit* model does not allow full control of the fitting parameters.^3^ The range of the viscosity of protein layers however correlates with reported values.^4^ (*) Some of the protein layers resulted in a very rigid layer and the viscoelastic properties could not be modeled correctly. Therefore, the raw dissipation and splitting of harmonics in Δ*D*/Δ*F* plots should be considered as indication for the viscoelastic properties.^5^


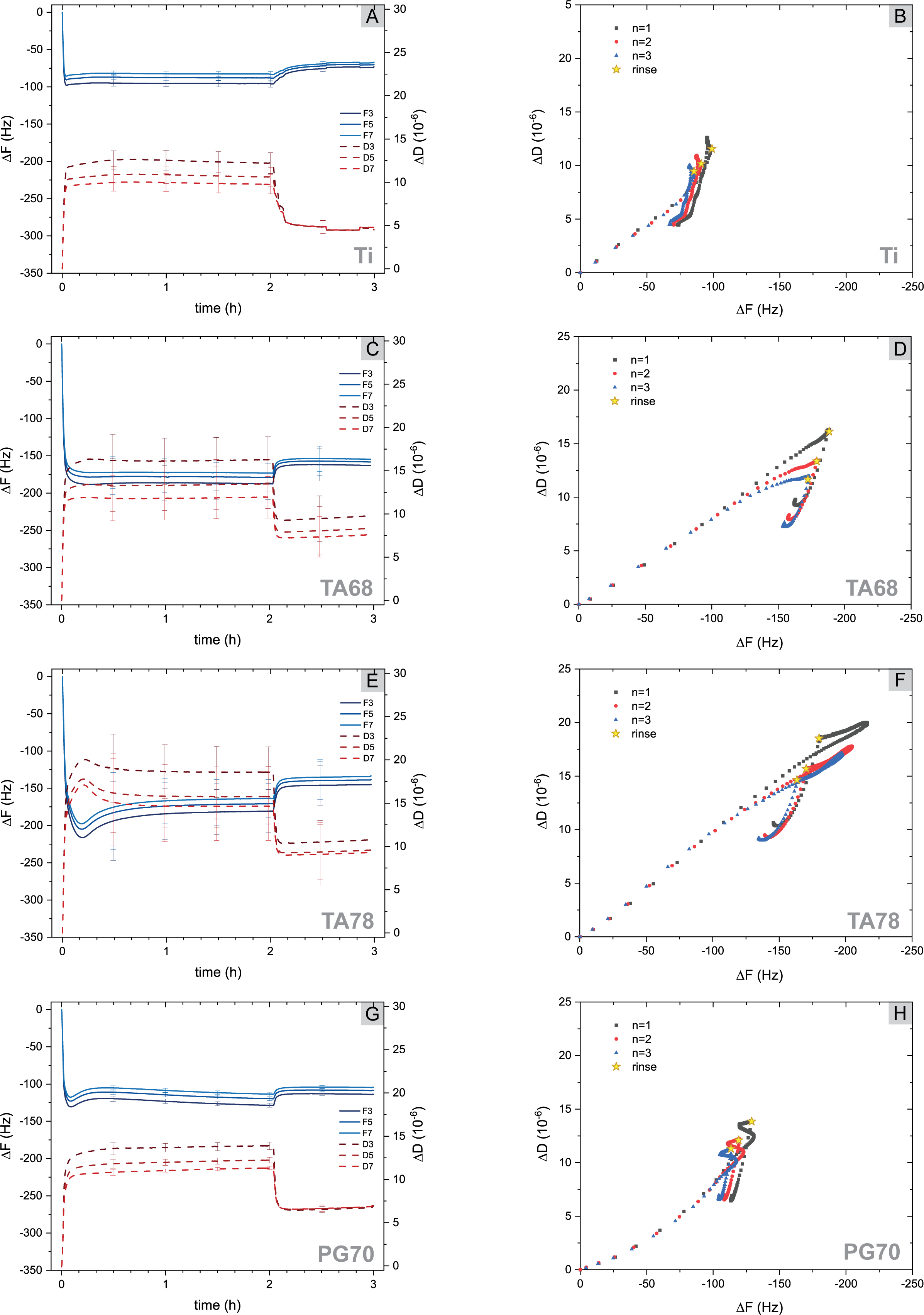


**Figure S4:** QCM-D raw data for modeled human plasma adsorption shown in Figure S3 and Figure 1 (n_e_ = 4). (A, B) Adsorption on bare Ti sensors, (C, D) TA coating formed for 2 h at pH = 6.8, (E, F) TA coating formed for 2 h at pH = 7.8, and (G, H) PG coating formed for 2 h at pH = 7.0. In Δ*D*/Δ*F* plots the viscoelastic properties vs the gain in mass can be derived from the slope of the curves for the individual harmonics (n).^6^ A high slope correlates to a more dissipative protein structure, whereas a low slope indicates a rigid layer. Stars mark the beginning of the rinsing step with PBS. PG 70 and TA 78 surfaces show s-and hook-shaped profiles before the rinsing step. This indicates a rearrangement of the protein layer during the adsorption. In contrast, the profile on Ti and TA 68 coatings was rather linear.


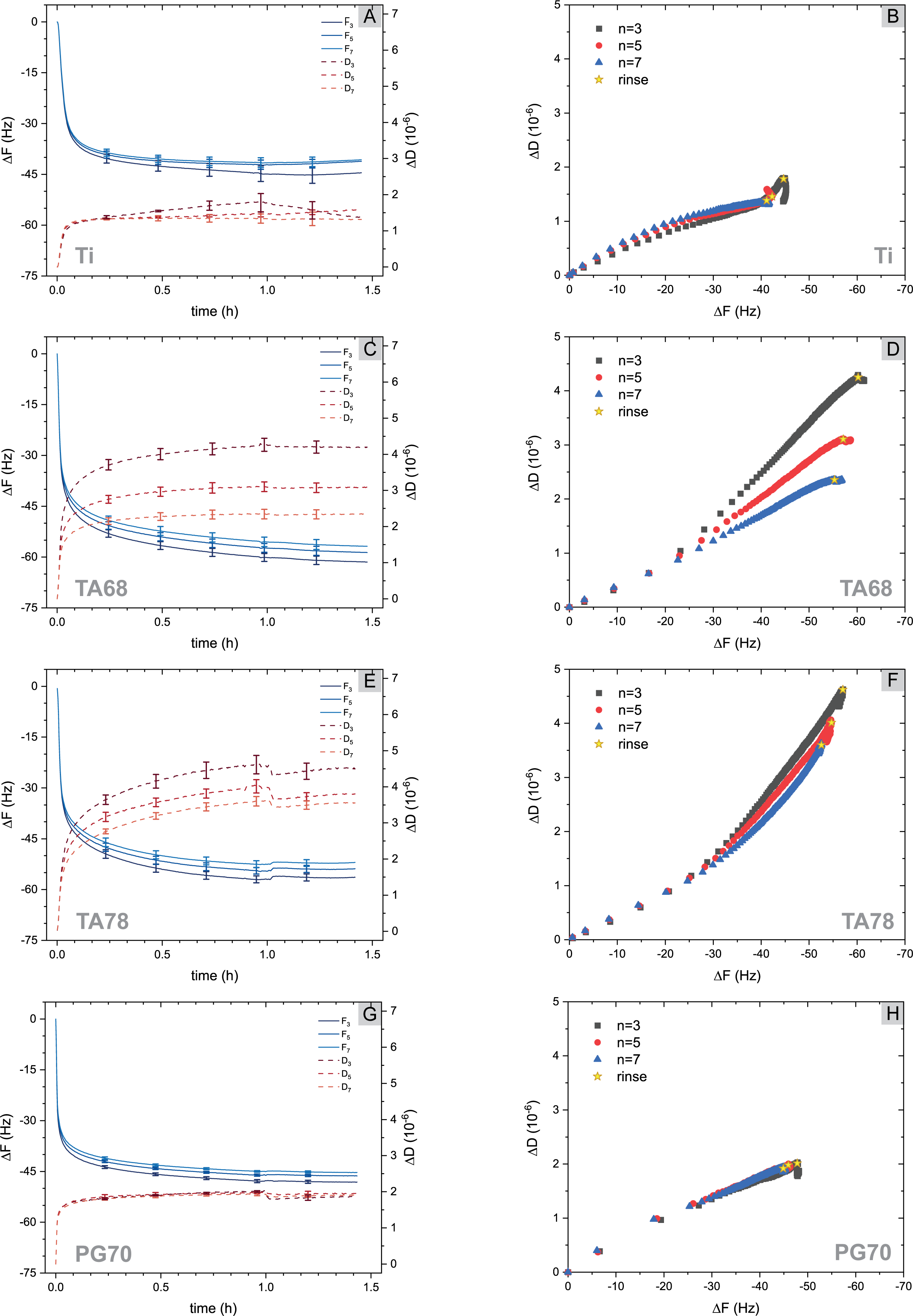


**Figure S5:** QCM-D raw data for modeled albumin adsorption shown in Figure S3 and Figure 1 (n_e_ = 4). (A, B) Adsorption on bare Ti sensors, (C, D) TA coating formed for 2 h at pH = 6.8, (E, F) TA coating formed for 2 h at pH = 7.8, and (G, H) PG coating formed for 2 h at pH = 7.0. In Δ*D*/Δ*F* plots the viscoelastic properties vs the gain in mass can be derived from the slope of the curves for the individual harmonics (n).^6^ A high slope correlates to a more dissipative protein structure, whereas a low slope indicates a rigid layer. Stars mark the beginning of the rinsing step with PBS. Adsorption of albumin followed a linear adsorption on all surfaces. On TA 68 and TA 78, splitting harmonics indicate a more rigid structure close to the surface. The larger initial spacing in Δ*D*/Δ*F* plots shows the faster adsorption kinetic for polyphenol modified surfaces.


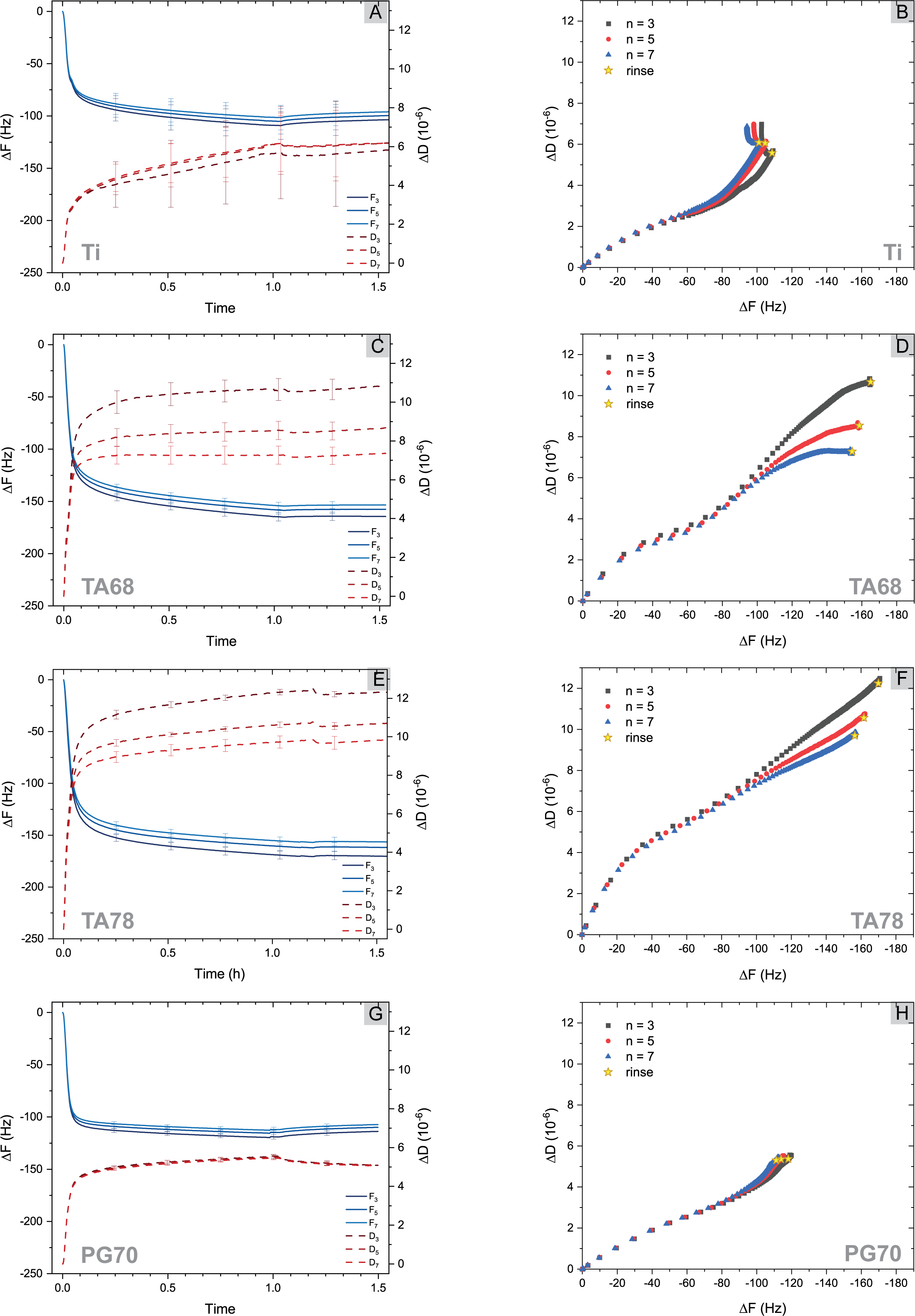


**Figure S6:** QCM-D raw data for modeled fibrinogen adsorption shown in Figure S3 and Figure 1 (n_e_ = 4). (A, B) Adsorption on bare Ti sensors, (C, D) TA coating formed for 2 h at pH = 6.8, (E, F) TA coating formed for 2 h at pH = 7.8, and (G, H) PG coating formed for 2 h at pH = 7.0. In Δ*D*/Δ*F* plots the viscoelastic properties vs the gain in mass can be derived from the slope of the curves for the individual harmonics (n).^6^ A high slope correlates to a more dissipative protein structure, whereas a low slope indicates a rigid layer. Stars mark the beginning of the rinsing step with PBS. Fibrinogen adsorbs in a multi-phase regime to the tested surfaces compared to albumin as Δ*D*/Δ*F* plots show. In the last phase, splitting harmonics indicate an inhomogeneous layer density on TA surfaces.


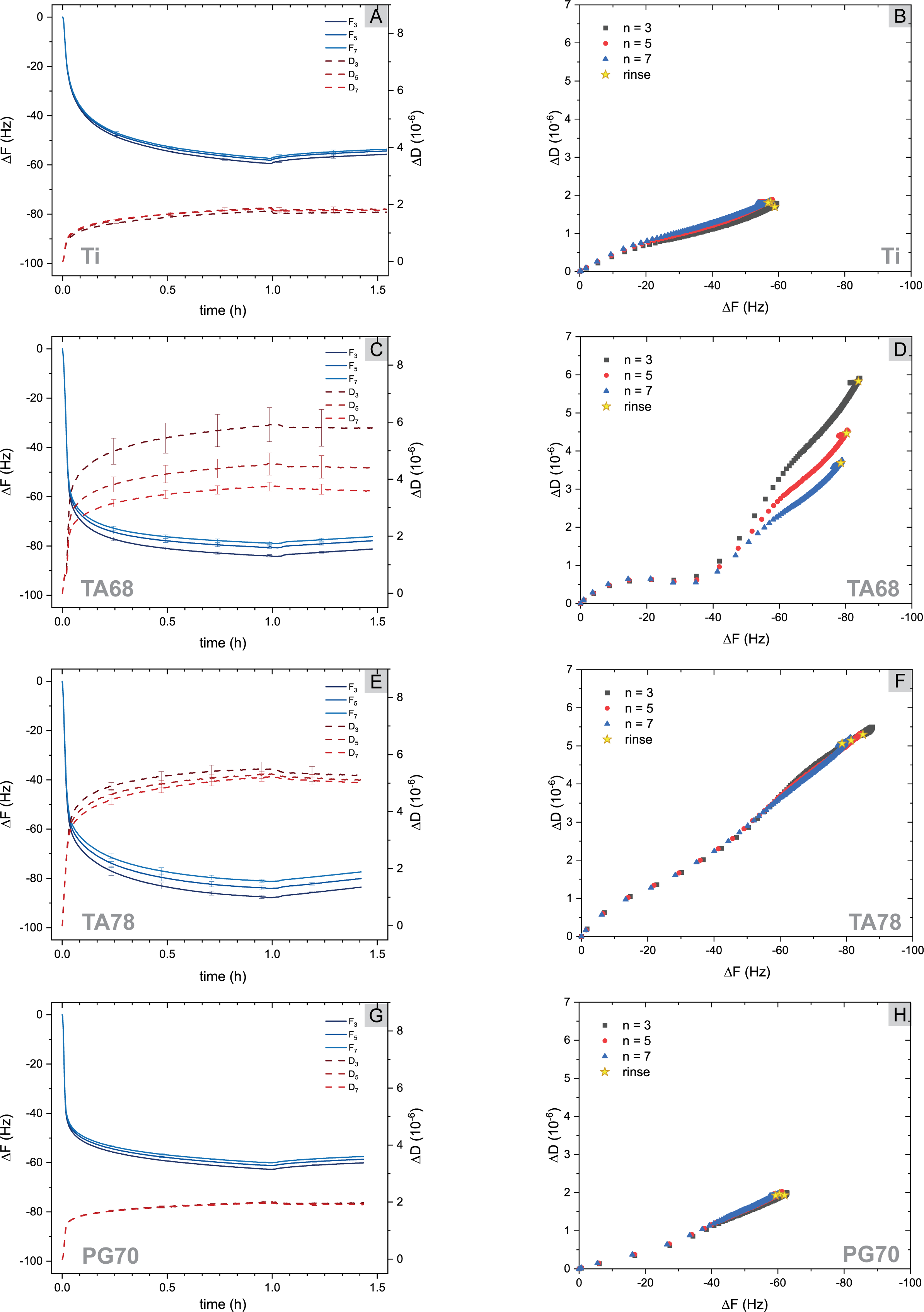


**Figure S7:** QCM-D raw data for modeled IgG adsorption shown in Figure S3 and Figure 1 (n_e_ = 4). (A, B) Adsorption on bare Ti sensors, (C, D) TA coating formed for 2 h at pH = 6.8, (E, F) TA coating formed for 2 h at pH = 7.8, and (G, H) PG coating formed for 2 h at pH = 7.0. In Δ*D*/Δ*F* plots the viscoelastic properties vs the gain in mass can be derived from the slope of the curves for the individual harmonics (n).^6^ A high slope correlates to a more dissipative protein structure, whereas a low slope indicates a rigid layer. Stars mark the beginning of the rinsing step with PBS. IgG adsorbs in a linear fashion on Ti and PG 70. In contrast, TA 68 and TA 78 shows altered adsorption behavior in Δ*D*/Δ*F* plots.


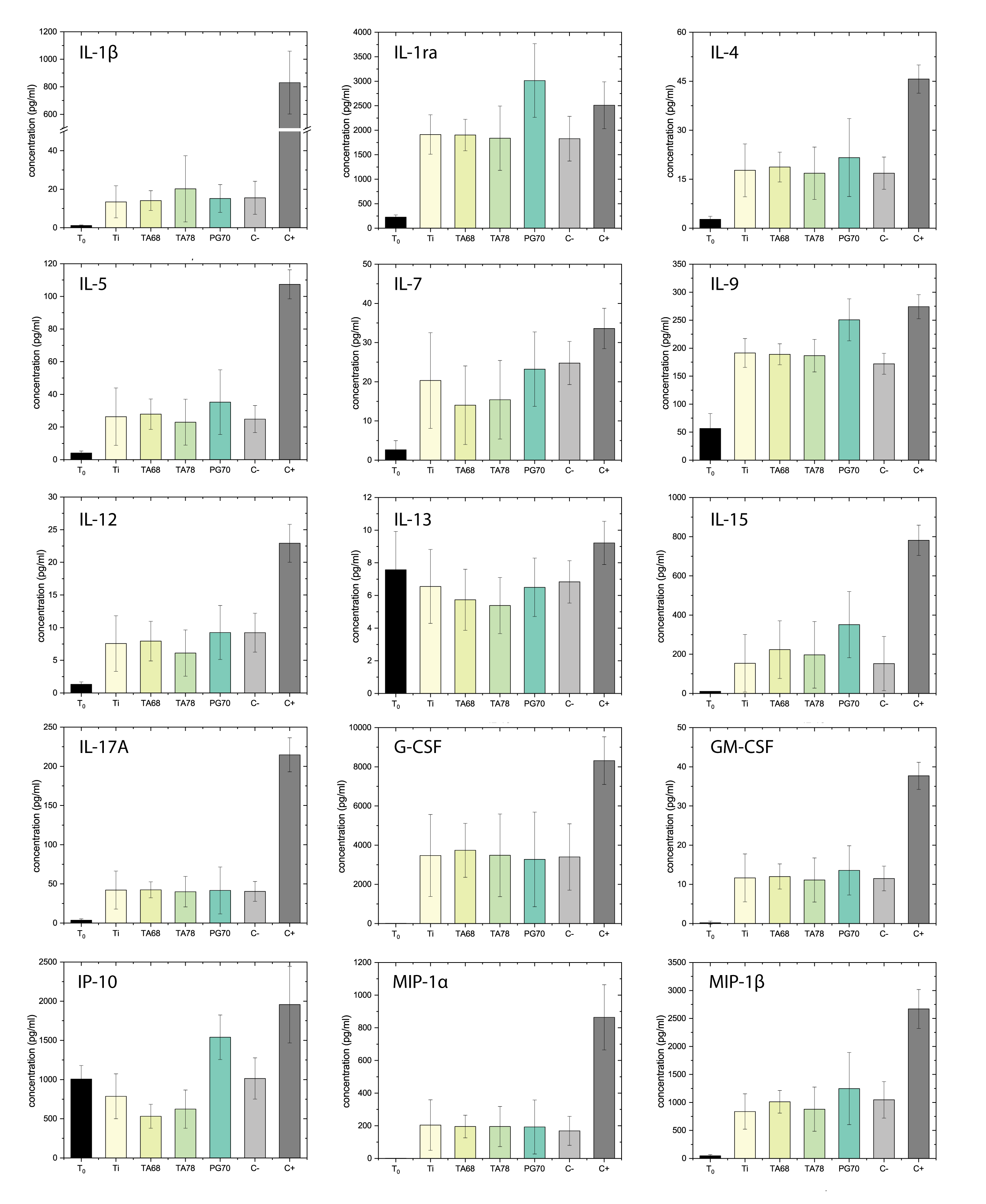


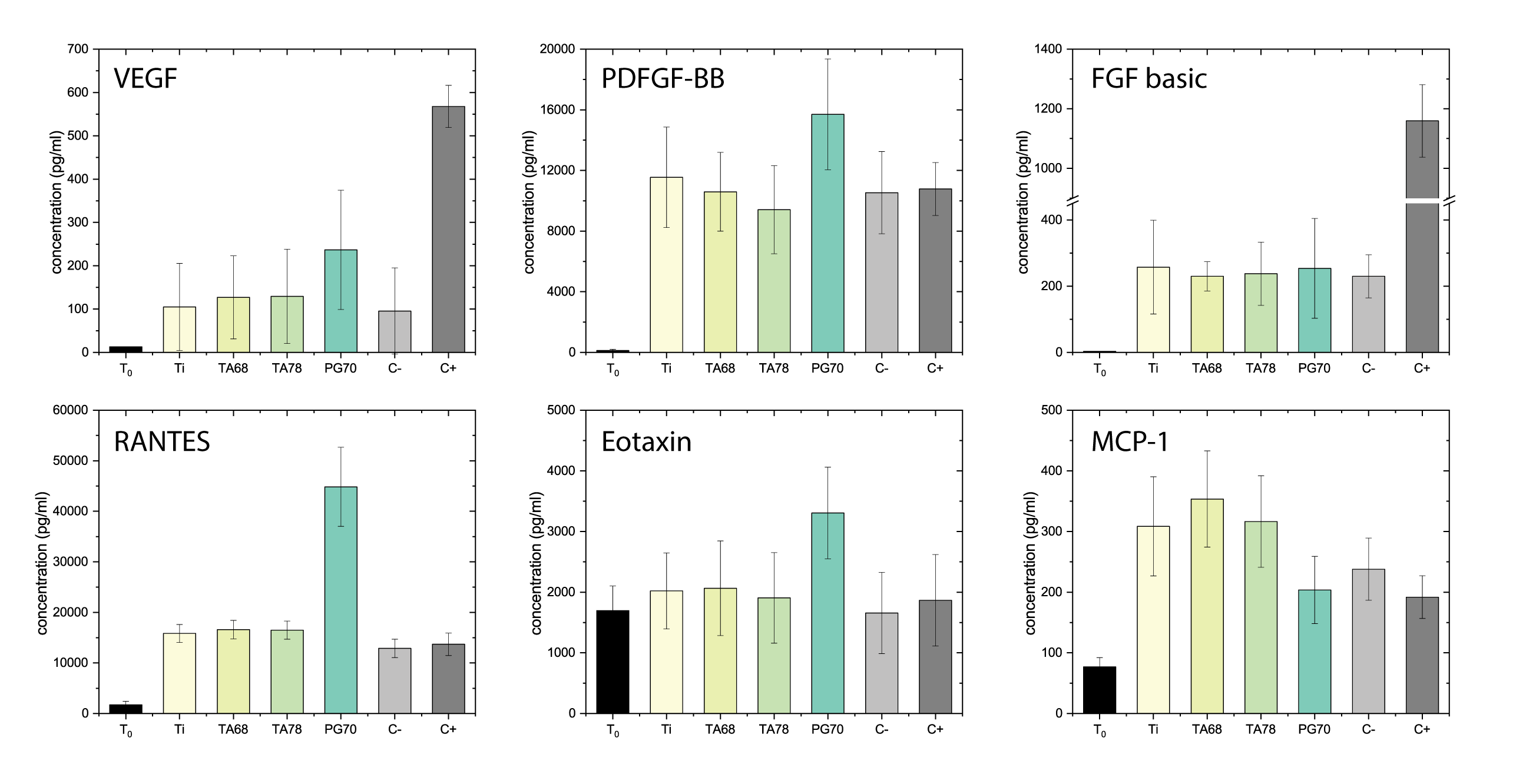


**Figure S8:** Released cytokine after incubation of Ti and polyphenols coated Ti surface in blood for 4 h. T_0_ denotes the cytokine baseline levels of 3 donors directly after blood donation. All samples were performed in triplicates for each donor (n_e_ = 9). C- denotes blood taken into polypropylene tubes with PBS added for volume control, whereas C+ denotes blood incubated with *E. coli*.


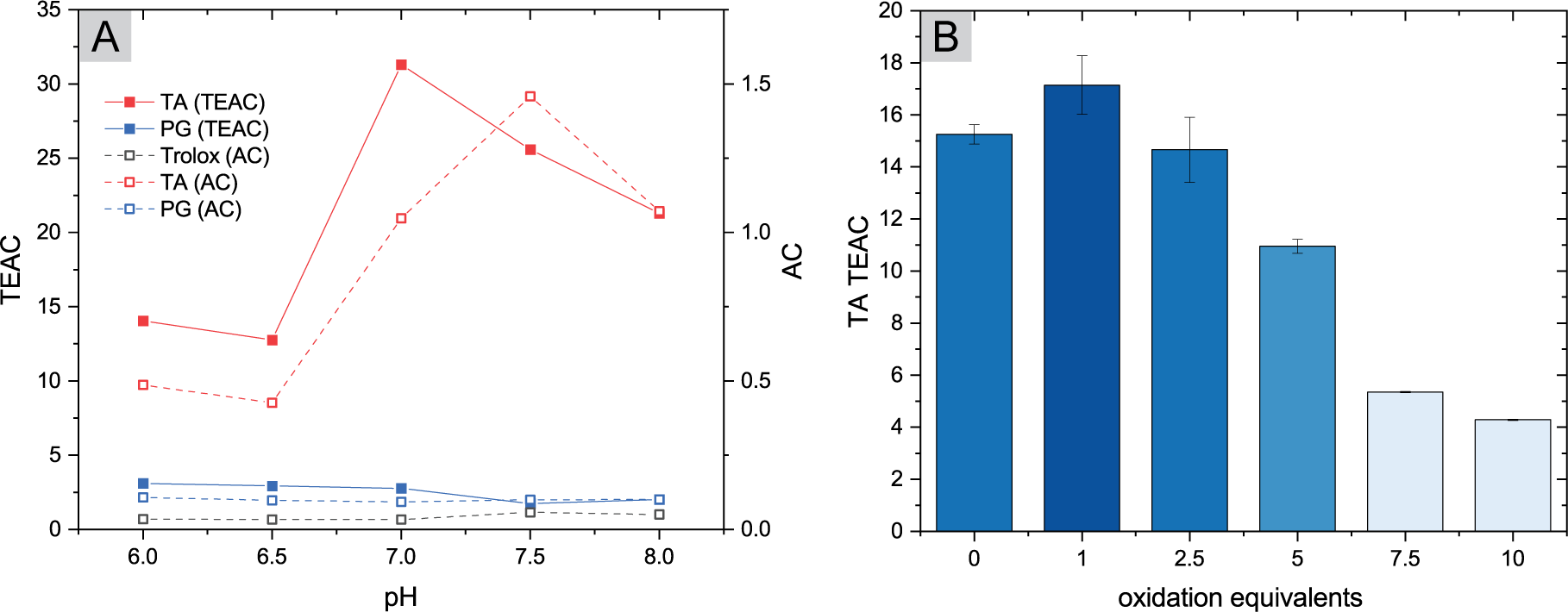


**Figure S9:** (A) TA and PG antioxidant capacity (AC) and trolox equivalent antioxidant capacity (TEAC) after 1h incubation in 10 mM phosphate buffer. Increasing the pH resulted in a slight increase in trolox activity. Since data for TA and PG has been normalized to trolox at each specific pH, a decrease of PG TEAC originated from the change in trolox AC. (B) Tannic acid antioxidant capacity expressed as TEAC after 1 h oxidation with given molar equivalents of sodium permanganate (n_e_ = 3). One equivalent corresponds to one gallic acid unit on TA. Since TA was assumed to consist of 10 units, 1 equivalent corresponds 10% of the TA concentration. Similar to the pH-based oxidation, 1 equivalent of sodium permanganate, increased the TEAC of TA. However, with further oxidation of TA the antioxidant capacity was reduced.


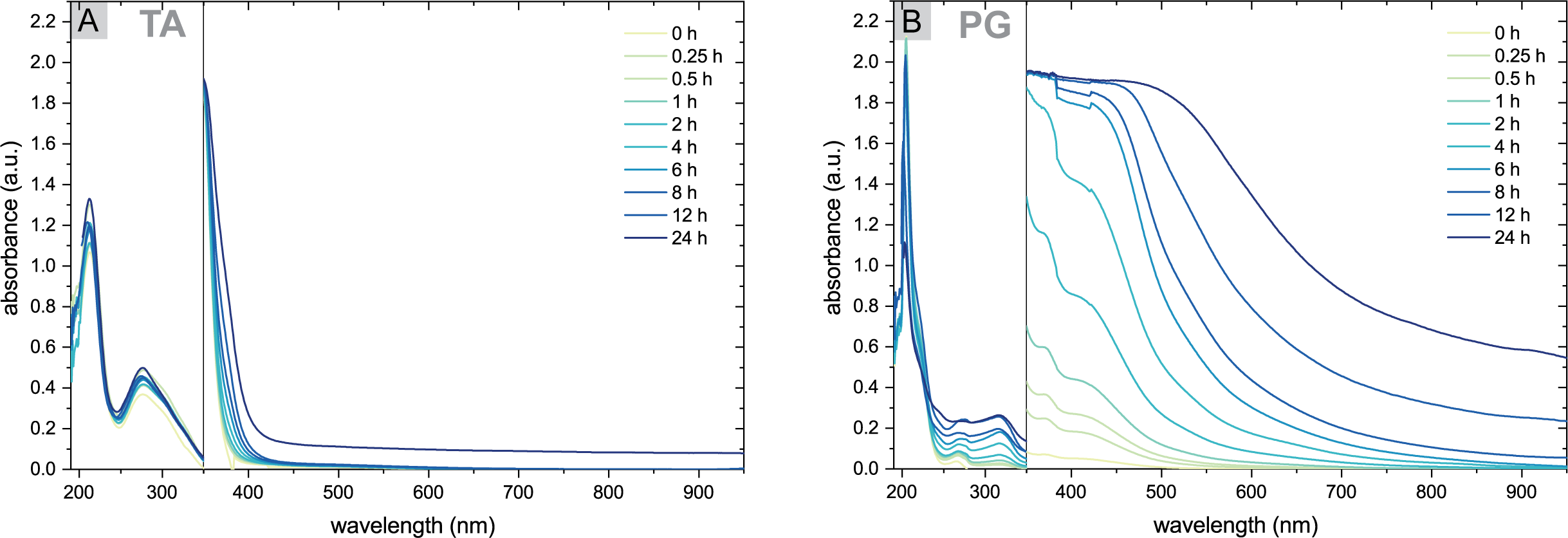


**Figure S10:** UV-vis spectrophotometric quantification of the oxidation of (A) TA and (B) PG. Polyphenols were dissolved at 1 mg/ml in 10 mM phosphate buffer at pH = 7.0 containing 150 mM NaCl. Due to their high UV absorbance, 100× diluted samples were scanned in the range between 190 nm ≤ λ ≤ 350 nm. Increase in adsorption for λ ≥ 250 nm indicated the oxidation at neutral pH.


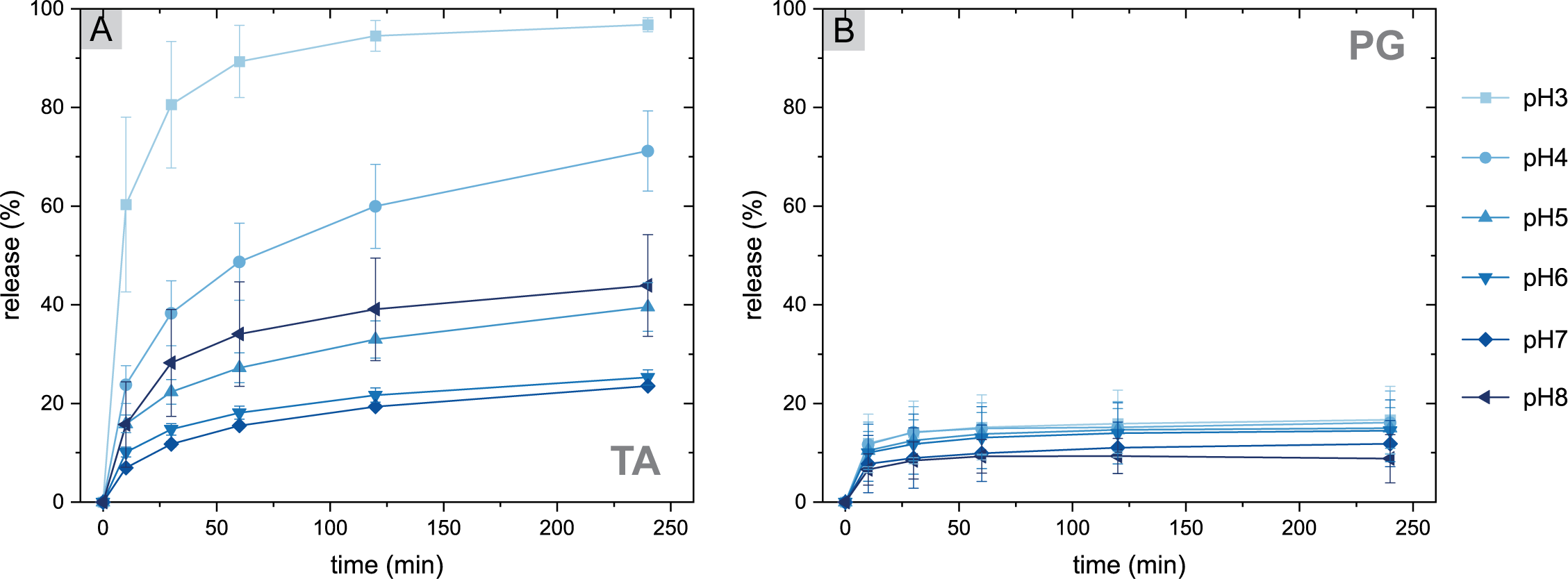


**Figure S11:** Release profile of (A) 2 h TA and (B) 10 h PG coatings in CPBS buffer at different pH. Coatings were formed on Ti sensors followed by a rinsing step before quantification of the dissolution of the layer by QCM-D (Figure S12A). Four individual experiments were conducted (n_e_ = 4). Mean values and SD in % and corresponding amount in μg/ml are given in Table S1.

**Table S1: Polyphenol release from coatings after 4 h at different pH measured by QCM-D (n_e_ = 4).**

| **pH** |  | Release after 4 h (%; μg/coin)^1^ | | | |  |
| --- | --- | --- | --- | --- | --- | --- |
|  | **TA** | | | **PG** | | |
| 3 | 96.5 ± 1.4 | | 8.5 ± 0.9 | 10.9 ± 3.5 | 0.09 ± 0.03 | |
| 4 | 71.1 ± 8.2 | | 7.1 ± 0.8 | 6.7 ± 5.0 | 0.05 ± 0.03 | |
| 5 | 37.5 ± 4.9 | | 2.6 ± 0.5 | 8.7 ± 1.0 | 0.07 ± 0.02 | |
| 6 | 23.1 ± 1.1 | | 2.1 ± 0.4 | 6.4 ± 1.8 | 0.05 ± 0.01 | |
| 7 | 21.5 ± 0.8 | | 1.8 ± 0.4 | 3.2 ± 1.1 | 0.03 ± 0.01 | |
| 8 | 43.4 ± 10.7 | | 3.8 ± 0.4 | 3.6 ± 3.8 | 0.04 ± 0.02 | |
| ^1^value calculated from Sauerbrey mass (ng/cm^2^) × coin area (104 mm^2^).  Note coatings were not deposited for 24 h compared to the Prussian Blue assay. | | | | | | |


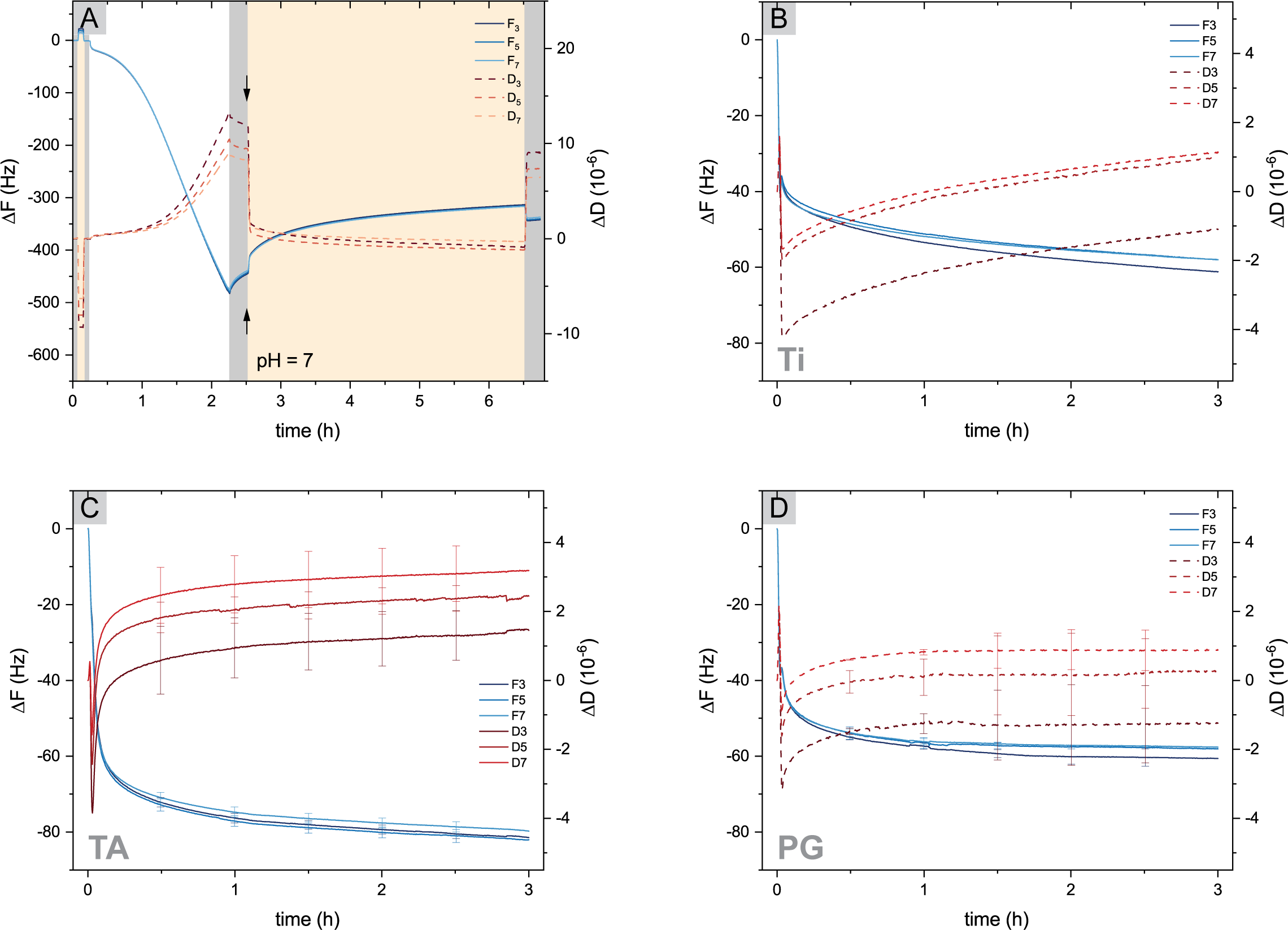


**Figure S12:** (A) Exemplary measurement setup for the formation of TA 78 coating and the release of molecules in PBS at pH = 7.0. Before the coating formation, the influence of buffers was controlled (HEPES in gray, PBS in orange). Subsequently, polyphenols were deposited in HEPES buffer. Before changing to PBS, a rinsing step with the coating buffer was conducted. (B-D) In contrast to desorption in PBS, no desorption was verified in DMEM (*n*_e_ = 3). Due to the protein and amino acid content of DMEM supplemented with 10% FBS, an increase in dissipation and decrease in frequency was observed. Since these changes were larger compared to Ti, we were not able to make a statement about the release of polyphenols.


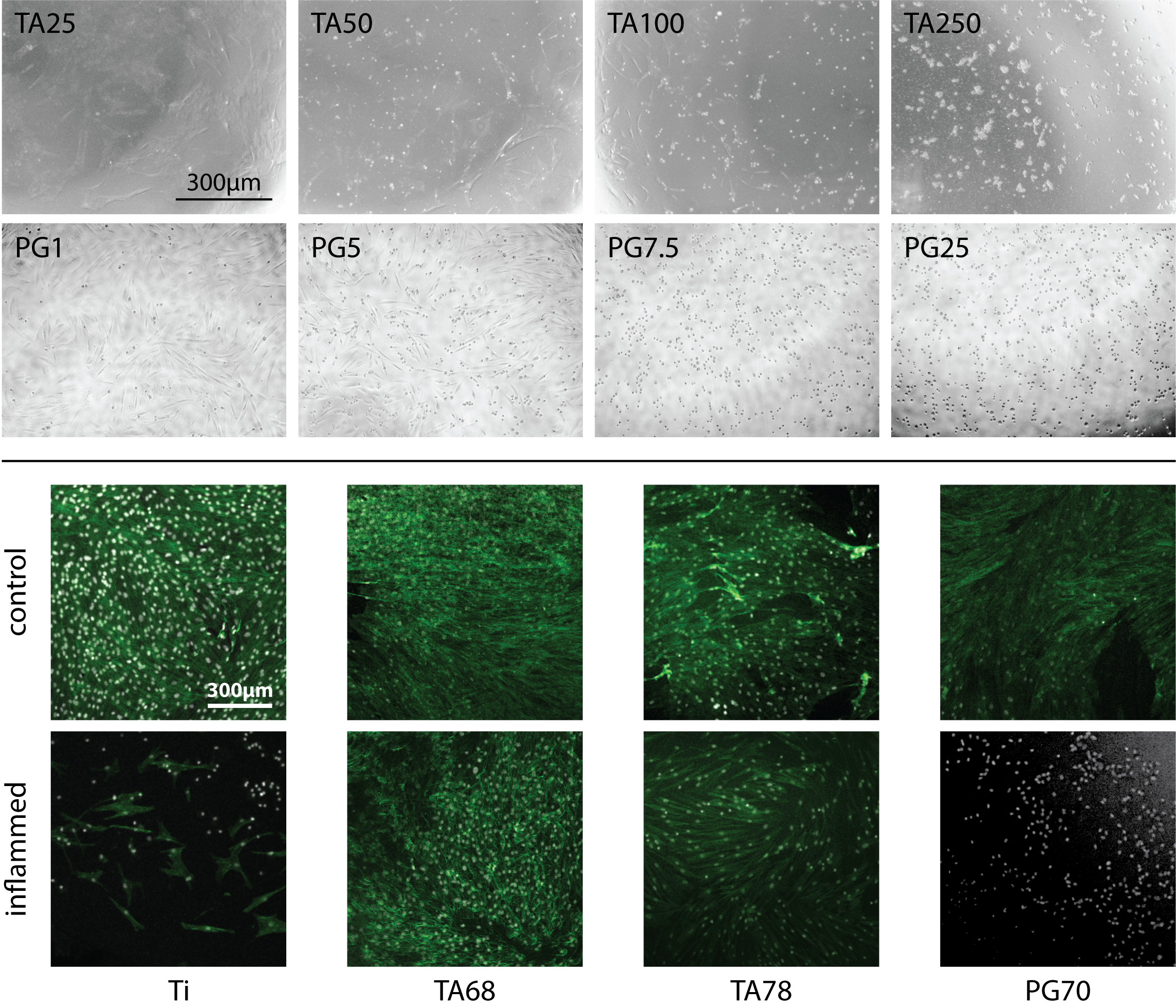
 **Figure S13:** Morphological changes of hGFs after incubation with TA and PG. Cells were initially allowed to adhere for 2 h before polyphenolic molecules were supplemented or inflammation was induced. *Top panel*: hGF cultured in DMEM on TCP with given amounts of dissolved polyphenols. For TA concentrations ≤ 25 μg/ml good cell adhesion with phenotypical spreading was observed after 24 h incubation. With increasing TA concentration, the cells adopted a round shape and did not adhere well to TCP. Similarly, dissolved PG showed phenotypical cell morphology for concentrations ≤ 5 μg/ml but round shape above this concentration. *Bottom panel*: hGFs seeded on coated Ti coins showed good adhesion and spreading after 48 h of incubation. Upon inflammation with LPS/IL-1β, hGFs showed a higher ratio of round morphology on Ti coins compared to TA 68 and TA 78 coated coins. On the PG coated coin, the phalloidin staining was rather weak and repeating the experiment showed that PG coatings appeared to quench the stain. A control experiment performed using PG-coated glass slides, which allowed viewing the cells with transmitted light, showed phenotypical regular shaped cells. Thus, we suppose there was a problem with the staining.


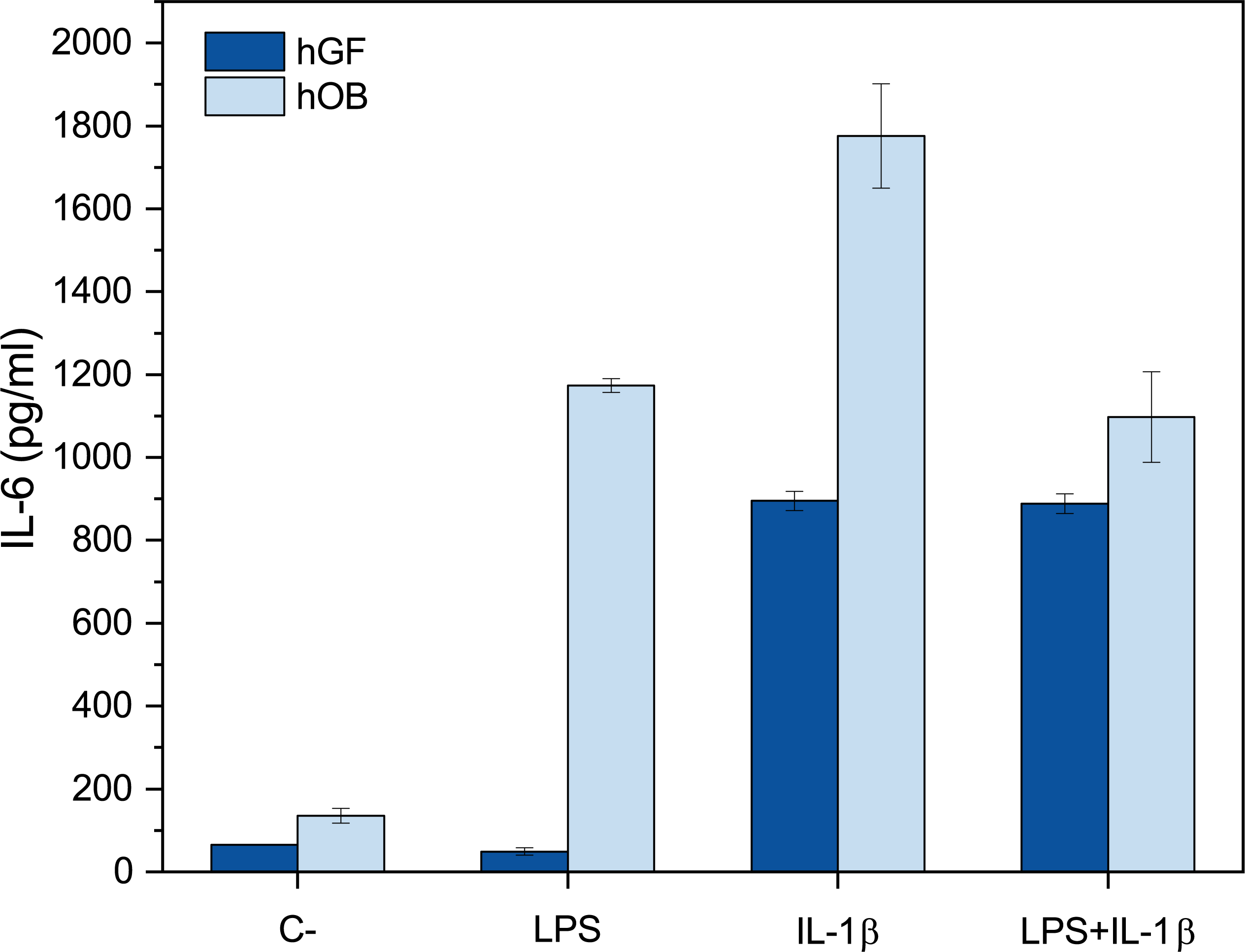


**Figure S14:** Reaction of hGFs to 1 μg/ml LPS derived from *P. gingivalis*, 1 ng/ml IL-1β, or both in combination. LPS and IL-1β were added to medium containing 1% FBS. Cells were incubated for 24 h. To control whether the effect was caused by inactive LPS, we subjected hOBs to the same conditions and obtained a positive inflammatory response upon exposure to LPS. Thus, we ruled out an effect of the LPS,^7^ but hypothesized that our hGFs were in some way resistant to LPS.^8^ Values are shown as mean ± SD (n_e_ = 3).


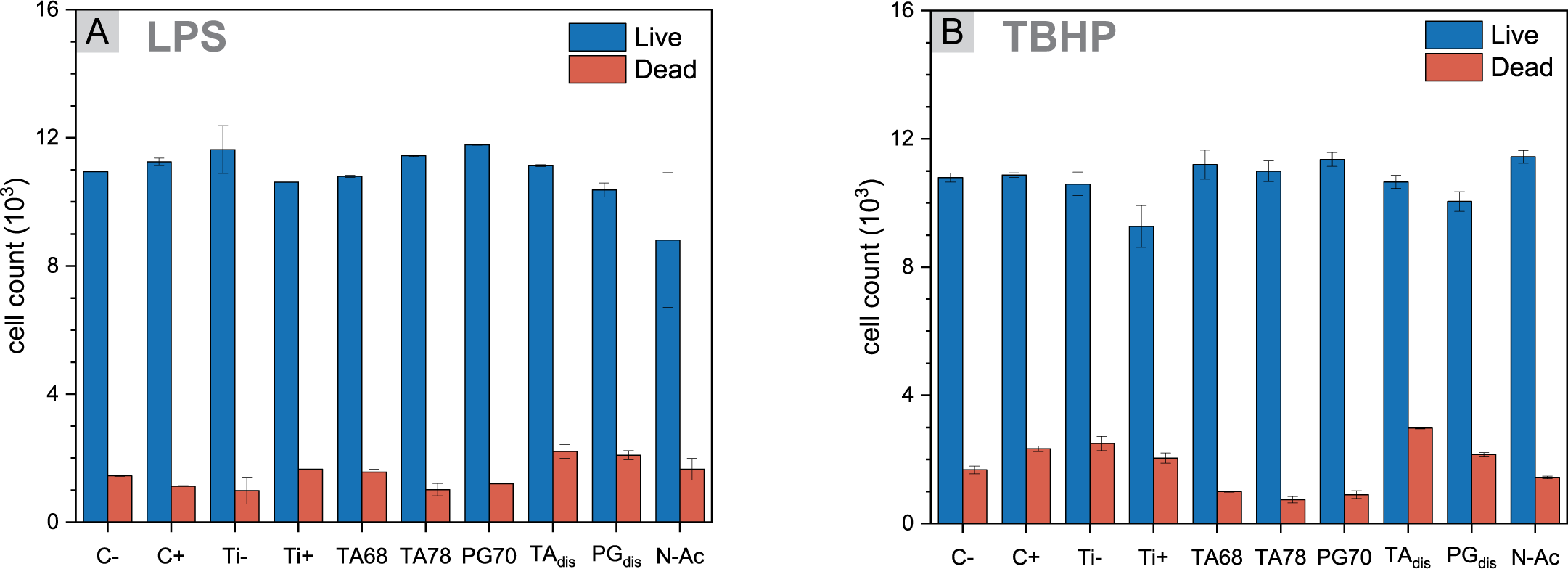


**Figure S15:** Number of live and dead cells stained with propidium iodide (PI) and counted by flow cytometry. All samples were seeded with 25×10^3^ cells per well and 20×10^3^ events were registered during the measurement. Irregular shaped cells and aggregates were excluded for analysis giving an overall similar number of cells between the groups. C+ and C- denote inflammed and not inflammed cells cultured on tissue culture plastic. Mean and standard deviation are reported for cells incubated on two individual surfaces. We did not stain for apoptosis since apoptotic cells are not considered to cause further inflammation compared to necrotic cells.^9^ For each group, two samples were prepared (n_e_ = 2) and 10k cells registered.


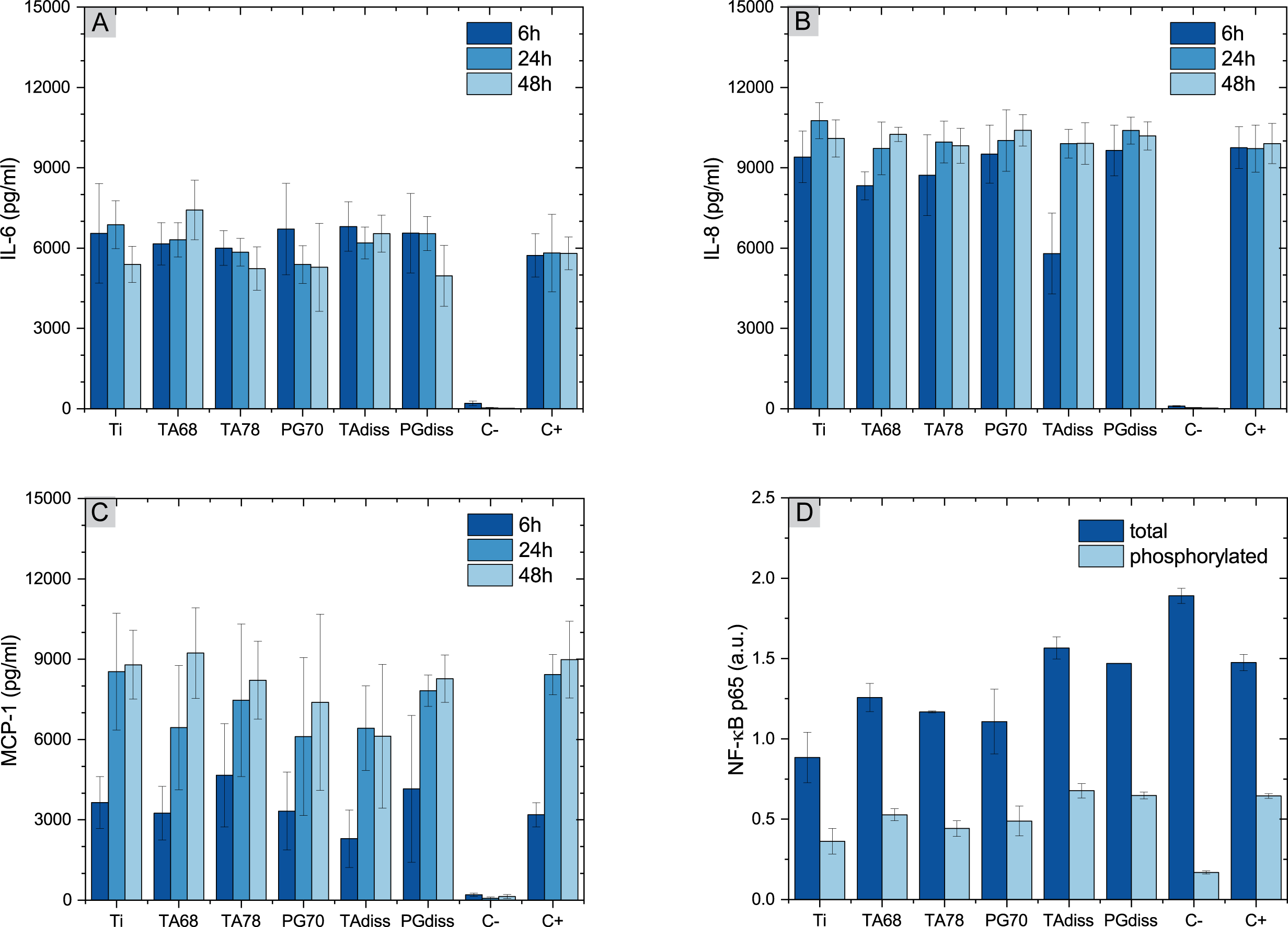


**Figure S16:** (A – C) Temporal expression of cytokines by hGFs (n_e_ = 6). Cells were inflamed with LPS/IL-1β and medium was analyzed after 6 h, 24 h, and 48 h for the expression of IL-6 (A), IL-8 (B), and MCP-1 (C) using a multiplex assay. Note that the upper limit of the standard curve for the cytokine quantification was about 10 ng/ml. (D) Total and phosphorylated NF-κB p65 levels were determined by ELISA 30 min after induction of inflammation (n_e_ = 3). Values are presented as mean ± SD.

# References

1 G. Bergseth, J. K. Ludviksen, M. Kirschfink, P. C. Giclas, B. Nilsson, T. E. Mollnes, *Mol. Immunol.,* 2013, **56**, 232.

2 M. V. Voinova, M. Rodahl, M. Jonson, B. Kasemo, *Phys. Scr.,* 1999, **59**, 391.

3 T. P. McNamara, C. F. Blanford, *Analyst,* 2016, **141**, 2911.

4 N. Weber, A. Pesnell, D. Bolikal, J. Zeltinger, J. Kohn, *Langmuir,* 2007, **23**, 3298.

5 G. A. McCubbin, S. Praporski, S. Piantavigna, D. Knappe, R. Hoffmann, J. H. Bowie, F. Separovic, L. L. Martin, *Eur. Biophys. J.,* 2011, **40**, 437.

6 M. Tagaya, *Polym. J.,* 2015, **47**, 599.

7 T. D. K. Herath, Y. Wang, C. J. Seneviratne, Q. Lu, R. P. Darveau, C.-Y. Wang, L. Jin, *J. Clin. Periodontol.,* 2011, **38**, 694.

8 M. J. Steffen, S. C. Holt, J. L. Ebersole, *Oral Microbiol. Immunol.,* 2000, **15**, 172.

9 K. L. Rock, H. Kono, *Annu. Rev. Pathol.,* 2008, **3**, 99.
